# Single-crystal orientation lithium for ultra-stable all-solid-state batteries

**DOI:** 10.1093/nsr/nwaf540

**Published:** 2025-12-01

**Authors:** Qidong Li, Likun Chen, Junyu Jiao, Yang Zhao, Suting Weng, Jun Zhao, Jiabin Ma, Yuhang Li, Genming Lai, Shichao Wu, Xufei An, Ke Yang, Jie Biao, Xing Cheng, Kai Shi, Jiaxin Zheng, Xuefeng Wang, Yongfu Tang, Ming Liu, Lele Peng, Wei Lv, Jun Lu, Feiyu Kang, Quan-Hong Yang, Yan-Bing He

**Affiliations:** Shenzhen All-Solid-State Lithium Battery Electrolyte Engineering Research Center, Institute of Materials Research (IMR), Tsinghua Shenzhen International Graduate School, Tsinghua University, Shenzhen 518055, China; Shenzhen All-Solid-State Lithium Battery Electrolyte Engineering Research Center, Institute of Materials Research (IMR), Tsinghua Shenzhen International Graduate School, Tsinghua University, Shenzhen 518055, China; School of Advanced Materials, Peking University Shenzhen Graduate School, Shenzhen 518055, China; Shenzhen All-Solid-State Lithium Battery Electrolyte Engineering Research Center, Institute of Materials Research (IMR), Tsinghua Shenzhen International Graduate School, Tsinghua University, Shenzhen 518055, China; Beijing Key Laboratory for New Energy Materials and Devices, Institute of Physics, Chinese Academy of Sciences, Beijing 100190, China; Clean Nano Energy Center, State Key Laboratory of Metastable Materials Science and Technology, Yanshan University, Qinhuangdao 066004, China; Shenzhen All-Solid-State Lithium Battery Electrolyte Engineering Research Center, Institute of Materials Research (IMR), Tsinghua Shenzhen International Graduate School, Tsinghua University, Shenzhen 518055, China; Shenzhen All-Solid-State Lithium Battery Electrolyte Engineering Research Center, Institute of Materials Research (IMR), Tsinghua Shenzhen International Graduate School, Tsinghua University, Shenzhen 518055, China; School of Advanced Materials, Peking University Shenzhen Graduate School, Shenzhen 518055, China; Nanoyang Group, Tianjin Key Laboratory of Advanced Carbon and Electrochemical Energy Storage, State Key Laboratory of Chemical Engineering, School of Chemical Engineering and Technology, Tianjin University, Tianjin 300072, China; Shenzhen All-Solid-State Lithium Battery Electrolyte Engineering Research Center, Institute of Materials Research (IMR), Tsinghua Shenzhen International Graduate School, Tsinghua University, Shenzhen 518055, China; Shenzhen All-Solid-State Lithium Battery Electrolyte Engineering Research Center, Institute of Materials Research (IMR), Tsinghua Shenzhen International Graduate School, Tsinghua University, Shenzhen 518055, China; Shenzhen All-Solid-State Lithium Battery Electrolyte Engineering Research Center, Institute of Materials Research (IMR), Tsinghua Shenzhen International Graduate School, Tsinghua University, Shenzhen 518055, China; Shenzhen All-Solid-State Lithium Battery Electrolyte Engineering Research Center, Institute of Materials Research (IMR), Tsinghua Shenzhen International Graduate School, Tsinghua University, Shenzhen 518055, China; Shenzhen All-Solid-State Lithium Battery Electrolyte Engineering Research Center, Institute of Materials Research (IMR), Tsinghua Shenzhen International Graduate School, Tsinghua University, Shenzhen 518055, China; School of Advanced Materials, Peking University Shenzhen Graduate School, Shenzhen 518055, China; Beijing Key Laboratory for New Energy Materials and Devices, Institute of Physics, Chinese Academy of Sciences, Beijing 100190, China; Clean Nano Energy Center, State Key Laboratory of Metastable Materials Science and Technology, Yanshan University, Qinhuangdao 066004, China; Shenzhen All-Solid-State Lithium Battery Electrolyte Engineering Research Center, Institute of Materials Research (IMR), Tsinghua Shenzhen International Graduate School, Tsinghua University, Shenzhen 518055, China; Shenzhen All-Solid-State Lithium Battery Electrolyte Engineering Research Center, Institute of Materials Research (IMR), Tsinghua Shenzhen International Graduate School, Tsinghua University, Shenzhen 518055, China; Shenzhen All-Solid-State Lithium Battery Electrolyte Engineering Research Center, Institute of Materials Research (IMR), Tsinghua Shenzhen International Graduate School, Tsinghua University, Shenzhen 518055, China; College of Chemical and Biological Engineering, Zhejiang University, Hangzhou 310027, China; Shenzhen All-Solid-State Lithium Battery Electrolyte Engineering Research Center, Institute of Materials Research (IMR), Tsinghua Shenzhen International Graduate School, Tsinghua University, Shenzhen 518055, China; Nanoyang Group, Tianjin Key Laboratory of Advanced Carbon and Electrochemical Energy Storage, State Key Laboratory of Chemical Engineering, School of Chemical Engineering and Technology, Tianjin University, Tianjin 300072, China; Shenzhen All-Solid-State Lithium Battery Electrolyte Engineering Research Center, Institute of Materials Research (IMR), Tsinghua Shenzhen International Graduate School, Tsinghua University, Shenzhen 518055, China

**Keywords:** all-solid-state lithium battery, single-crystal orientation lithium metal, crystal orientation regulation, interfacial stability, long cycling performance

## Abstract

All-solid-state lithium (Li) metal batteries (ASLMBs), particularly with inorganic solid electrolytes, possess both high energy density and high safety. However, their practical application is still being severely impeded by Li dendrite formation as a fundamental but unclear issue. Here, we reveal that the anisotropic exfoliation of polycrystal Li metal due to different energies required for Li atom stripping from various Li crystal planes leads to the formation of voids upon cycling, which is the intrinsic cause for the formation of Li dendrites and interfacial cracks. We thereafter precisely tune the polycrystal Li metal to <110>-oriented single-crystal Li metal using a lattice-matching template of Li_2_Ga (131) interface. During the stripping process of <110>-oriented single-crystal Li, the unstripped surface Li atoms at the Li (110) plane present lower stripping energy than those of the fresh layers, which ensures layer-by-layer Li stripping/plating and avoids Li void formation to fundamentally suppress the Li dendrite generation during long cycling. The ASLMBs using <110>-oriented single-crystal Li have ultralong stability of over 10 000 cycles at 25°C. Our results establish that regulating the crystal orientation of Li metal is a basic and practical solution for solving the dendrite formation problem and pushing forward the final real applications of ASLMBs.

## Teaser

This work precisely tunes the polycrystal Li metal to <110>-oriented single-crystal Li metal, which avoids Li dendrite generation to achieve ultralong cycling stability of all-solid-state lithium metal batteries.

## INTRODUCTION

All-solid-state lithium (Li) metal batteries (ASLMBs), particularly with inorganic solid electrolytes (ISEs), are believed to be an ideal replacement for the present liquid electrolyte-based Li-ion batteries mainly due to the intrinsic safety of the ISE and the high energy density given by the high specific capacity of the Li metal anode [1–6]. However, ASLMBs face huge challenges before their practical use is realized, including the unsolved problem of dendrite formation and rapid degradation of the interface with the electrolyte during long cycling [1,7–9]. Local high currents at the anode/electrolyte interfaces are considered the main cause for the nucleation of Li dendrites in ISEs, and these are mainly ascribed to degradation of the interface to form Li voids during long cycling [1,2,7]. Interface degradation and Li voids easily arise during Li stripping, even with a carefully modified interface [10]. Unfortunately, the mechanism for such deterioration remains unclear.

The vast majority of crystal materials feature different properties in different directions, and any given crystal planes have their own surface energies, and this is also true for Li metal, which has a polycrystal structure [11–15]. Various growth directions of Li dendrites have been

observed and those with <111> and <112> orientations have much higher internal stresses than one with a <110> orientation [11,16]. This greatly influences Li dendrite growth in Li metal batteries with liquid electrolytes [13]. The Li crystal planes at different orientations have fundamentally different thermodynamic properties. Specifically, the energy required to strip/plate Li atoms from/on different crystal planes varies drastically, which alters the Li stripping/plating behavior dramatically. Accordingly, the stripping/plating of differently oriented crystals in the Li anode may be a fundamental reason for the degradation of the interface with an ISE, and this has not previously been taken into account [17–20]. Based on this analysis, tuning the polycrystal Li to a <110>-oriented single-crystal Li (denoted as Li <110> orientation) and meanwhile maintaining this orientation during long cycling is likely an ultimate solution towards solving the interface deterioration and Li dendrite growth for ASLMBs capable of long cycling, but which has remained a highly elusive challenge throughout the field.

**Figure 1.**
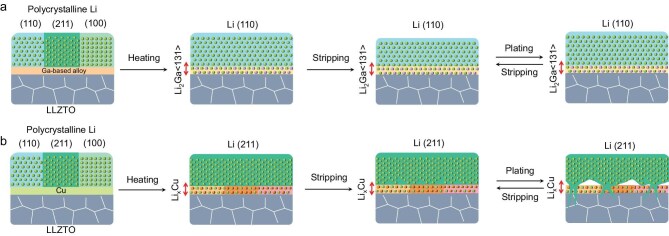
Schematic of how to control the oriented single-crystal Li and the cycling of Li metal with different crystal orientations. (a) <110>-Oriented single-crystal Li and layer-by-layer stripping/plating behavior regulated by a lattice matched Li_2_Ga (131) interface. (b) <211>-Oriented single-crystal Li and with uneven stripping/plating behavior regulated by a Li_x_Cu alloy interface.

In this work, we reveal that the multi-orientating Li stripping of various Li crystal planes in a polycrystal Li metal owing to different Li stripping energy is the root cause of the interface degradation and Li dendrite generation. Based on this, we achieve controlled layer-by-layer Li stripping/plating by a <110>-oriented single-crystal Li that is regulated using a lattice-matching Li_2_Ga (131) template. The <110>-oriented single-crystal Li has an extremely stable structure during long cycling, which avoids the formation of voids, and finally eliminates the generation of Li dendrites at the interface (Fig. 1a), whereas other oriented single-crystal Li such as the <211>-oriented single-crystal Li are more likely to strip/plate perpendicular to the planes and form voids (Fig. 1b), which causes Li dendrite growth. The Li–Li symmetrical battery using <110>-oriented single-crystal Li has a cycle life as long as 9000 h at 0.1 mA cm^−2^ and 3000 h at 0.5 mA cm^−2^. Moreover, when paired with an LiFePO_4_ (LFP) cathode, the as-prepared ASLMBs have a high-capacity retention of 92% after 6000 cycles at 3C and 25°C. Our work proves that controlling the single-crystal orientation of the Li metal anode is a brand-new strategy to solve the problems of dendrite growth and interface deterioration in ASLMBs.

## RESULTS

### Precise control of the <110>-oriented single-crystal Li

**Figure 2.**
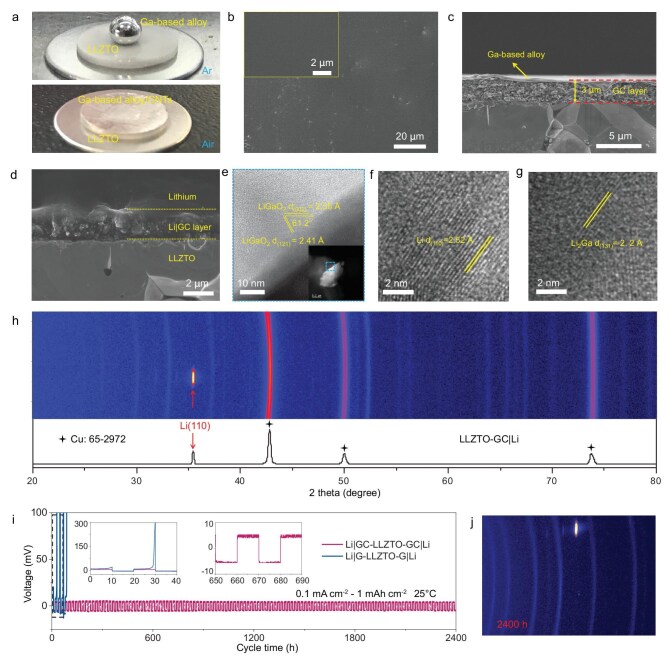
Construction of GC|Li on LLZTO to regulate the <110>-oriented single crystal Li. (a) Infiltrating behavior of GBA and GC layer on the LLZTO surface. (b and c) Top surface and cross-section SEM images of the LLZTO-GC. (d) Cross-section SEM image of LLZTO-GC|Li. (e–g) Cryo-HRTEM images of LLZTO-GC|Li. (h) 2D-XRD and corresponding integral 1D-XRD pattern of LLZTO-GC|Li. (i) Cycling performance of Li|GC-LLZTO-GC|Li and Li|G-LLZTO-G|Li at 0.1 mA cm^−2^ with an areal capacity of 1 mAh cm^−2^ at 25°C. (j) 2D-XRD of LLZTO-GC|Li after 2400 h operation.

A mixture containing gallium (Ga)-based alloy (GBA) and carbon nanotubes (CNTs) with a diameter of 40 nm was coated on a Li_6.4_La_3_Zr_1.4_Ta_0.6_O_12_ (LLZTO) sheet in air to form a flat GBA/CNT (GC) interface layer with uniform morphology (Fig. 2a and b and Fig. S1). A cross-section scanning electron microscopy (SEM) image shows that the layer has a thickness of around 3 μm and a distinct layer structure (Fig. 2c and Fig. S2a). A GBA oxide layer including Ga_2_O_3_, In_2_O_3_ and SnO_2_ was formed on the bottom of the GC layer as shown by X-ray photoelectron spectroscopy (XPS) (Fig. S2b and S2c) [21,22], which has intimate contact with the LLZTO sheet (Fig. 2c and Fig. S2a). This GBA oxide layer was generated as a result of the partial oxidation of GBA when exposed to air [23]. The CNTs as a framework are uniformly dispersed in the GC layer (Fig. S2a). A thin fluid GBA flat alloy film without oxidation was on top of the GC layer, as shown by SEM and XPS spectra (Fig. S2b) [21,24]. In addition, energy dispersive spectroscopy (EDS) maps show that Ga, In and Sn are evenly distributed in the GC layer (Fig. S2d–S2i), whereas a discontinuous and uneven layer was formed after coating a pure GBA layer (G layer) on LLZTO in air (Fig. S3), indicating that the CNTs improve the dispersion and homogeneity of the GC layer.

Transmission electron microscopy (TEM) images of LLZTO coated with a GBA layer were collected to identify the composition and microstructure of the GC layer (Fig. S4a–S4c). The LLZTO was tightly covered by a GBA oxide layer, which caused it to change color from white to black (Fig. S4a–S4c). A high-resolution TEM (HRTEM) image of the GBA oxide layer has lattice spacings of 2.41 and 2.69 Å, which are a good match with the (−310) and (−111) planes of Ga_2_O_3_ (JCPDF#41-1103), respectively (Fig. S4d). At the same time, a small number of unoxidized hemispherical GBA droplets remained on top of the Ga_2_O_3_ particles. These results prove that the GBA oxides play a bridging role between the GBA and LLZTO.

Li plates were melted on the LLZTO surface with GC and G layers at 300°C to construct the interface. Owing to the excellent fluidity of the GBA thin film on top of the GC layer, compact Li|GC and Li|G interfaces are formed, which make excellent contact with the LLZTO (Fig. 2d and Fig. S5a−S5d), whereas the LLZTO plate without interface modification makes poor contact with the molten Li metal (Fig. S5e). We also examined the XPS of the Li|GC layer at different positions to identify its components (Fig. S6). The Ga 2p3/2 spectrum of the Li|GC layer close to LLZTO has two peaks located at 1117.5 and 1116.2 eV, which are respectively assigned to LiGaO_2_ and Li_2_Ga [22]. The Ga in the Li|GC layer close to the Li metal changes to solid Li_2_Ga alloy after the reaction between the thin fluid GBA plate film and the Li metal at 300°C. Cryo-TEM and EDS elemental mapping of LLZTO-GC|Li confirm the uniform distribution of each element in the Li|GC interface (Fig. S7). Cryo-HRTEM shows that the Li|GC interface mainly consists of solid LiGaO_2_, Li_2_Ga, Li_2_O, Li_7_Sn_2_ and Li_13_In_3_, and that the phase in contact with the LLZTO is LiGaO_2_ because of a reaction between the GBA oxide layer, including Ga_2_O_3_, In_2_O_3_ and SnO_2_, and Li metal at 300°C (Fig. 2e–g and Fig. S8).

2D X-ray diffraction (XRD) was used to analyze the crystal orientations of the original Li plate and the recrystallized plate on different substrates, including LLZTO-GC, LLZTO-G and LLZTO after melting at 300°C. The original Li plate consists of typical polycrystals with <110>, <200>, <211> and <220> crystal orientations, while the recrystallized Li metal on LLZTO tends to form a single crystal with a random orientation due to no interfacial reaction with the Li metal (Figs S9 and S10). Thus, although the Li metal has a body-centered cubic structure, the Li (110) plane, as the closest packed plane with the lowest energy, is not preferentially formed as expected [25,26]. However, Li metal recrystallized on LLZTO modified by a GC layer (LLZTO-GC) has only one strong Li <110> crystal orientation that is perpendicular to the interface (Fig. 2h). In sharp contrast, the uneven G layer in LLZTO-G does not do this (Fig. S11). Thus, the GC layer with a uniform and smooth surface is critically important for the precise regulation of <110>-oriented single-crystal Li. The arrangement of the crystals in Li metal has a great influence on its physical and chemical properties, such as the mechanical properties [16]. It has been proved that the Li crystal with <110> orientation has a small yield strength and elastic modulus [11], which is quite beneficial for the protection of the ISEs in ASLMBs. Furthermore, we found that the other phases, such as In, Sn and their oxides, are crucial for increasing the interface wettability of the LLZTO by the GC layer (Fig. S12), and the CNTs help construct a flat and stable interface by significantly improving the mechanical strength of the GC layer, up to 863 N from the original 393 N (Figs S13 and S14). All these ensure a single-crystal orientation of the Li (110) plane parallel to the interface, giving excellent cycling stability (Fig. S15).

The cycling performances of Li|GC-LLZTO-GC|Li and Li|G-LLZTO-G|Li batteries at a current density of 0.1 mA cm^−2^ and a high area capacity of 1 mAh cm^−2^ were tested to examine the effect of Li crystal orientation on their cycling stability. Remarkably, the Li|GC-LLZTO-GC|Li cell had excellent cycling stability with no short-circuiting, even after 2400 h cycles (Fig. S16). Its stripping/deposition overpotential remained at about 5 mV and remained constant during cycling (Fig. 2i). In sharp contrast, the overpotential of a Li|G-LLZTO-G|Li battery using Li metal with only partial <110> orientation shows an increasing overpotential during the first stripping cycle and increased greatly in subsequent cycles (Fig. 2i, inset), which indicates a gradual degradation of the interface in LLZTO-G|Li at high area stripping capacity [10]. 2D-XRD shows that the <110>-oriented single-crystal Li in LLZTO-GC|Li is well maintained over 2400 h cycles of a symmetrical battery (Fig. 2j and Fig. S17).

### Mechanism for the control of <110>-oriented single-crystal Li by GC layer

**Figure 3.**
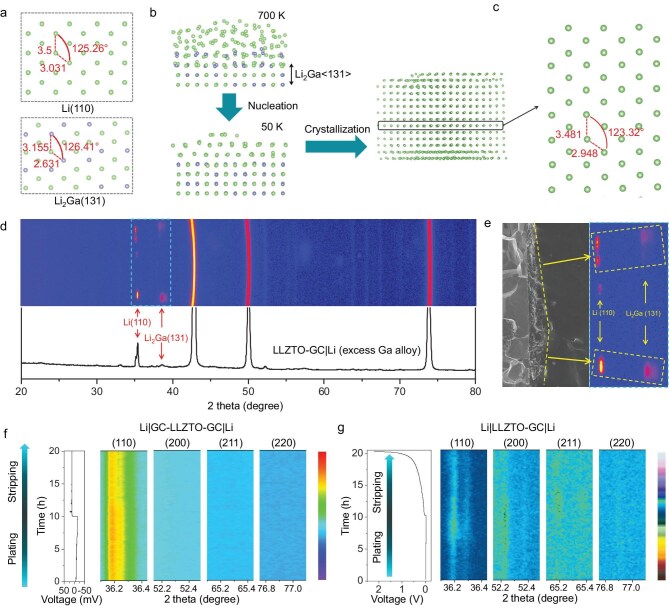
Regulation mechanism of the <110>-oriented single-crystal Li using a GC layer. (a) Atomic arrangement of Li (110) and Li_2_Ga (131) crystal planes. (b) Simulation of the melting/crystallization process of Li metal on the Li_2_Ga (131) crystal plane. (c) Atomic arrangement of Li metal after crystallization on the Li_2_Ga (131) crystal plane. (d) 2D-XRD and the corresponding integrated 1D-XRD pattern of LLZTO-GC|Li with excess GBA. (e) 2D-XRD signal of Li (110) and Li_2_Ga (131) crystal planes in LLZTO-GC|Li with excess GBA. (f and g) *In situ* 1D-XRD of Li|GC-LLZTO-GC|Li and Li|LLZTO-GC|Li cells during plating and stripping at 0.1 mA cm^−2^ for 10 h.

Based on the XPS and cryo-TEM results, it was found that although the GC layer is polycrystalline after its reaction with Li metal, the Li_2_Ga alloy layer formed on the GC layer directly contacts the Li metal anode. The Li_2_Ga (131) plane is a close-packed plane with the lowest surface energy and is preferentially formed on the top of the polycrystal GC buffer layer by a reaction between liquid Ga metal and Li metal at 300°C (Figs S6 and S18; Table S1). By comparing the crystal structure of Li_2_Ga with that of Li metal, it is seen that the atomic arrangement of the Li_2_Ga (131) crystal plane is very similar to that of the Li (110) crystal plane, with only a minor difference in the bond lengths (Fig. 3a). Quantitatively, the lattice mismatch between Li_2_Ga (131) and Li (110) is calculated as 8.9% (Fig. S19), which is much lower than typical values reported for epitaxial growth (usually below ∼15%), suggesting that the Li_2_Ga (131) and Li (110) planes form a lattice matching a semi-coherent interface to induce Li epitaxial growth [27]. Also, the flat liquid GBA film on the GC layer causes the Li_2_Ga (131) plane formed to be parallel to the interface.

We simulated the melting/crystallization of Li metal on Li_2_Ga (131) and found that the arrangement of Li atoms gradually changes from amorphous to ordered crystal as the temperature decreases (Fig. 3b). After crystallization, the bond lengths and angles between atoms in the Li crystal plane parallel to Li_2_Ga (131) are very close to those in Li (110) (Fig. 3c). These results prove that Li_2_Ga (131) acts as a template for the molten polycrystal Li to recrystallize in a <110>-oriented single-crystal Li. To verify this result, we increased the thickness of the interface layer by increasing the amount of GBA in it so that its phase information can be detected by XRD (Fig. S20). Figure 3d and e shows that diffraction patterns of Li_2_Ga (131) and Li (110) with the same orientation appear simultaneously after increasing the amount of GBA in the interface layer, which matches our simulation results very well, although the excess GBA causes it to aggregate (Fig. S20), which results in the formation of an imperfect Li <110> crystal orientation (Fig. 3e). Both the calculations and experimental results confirm that the directional crystallization of Li in a <110> orientation in LLZTO-GC|Li is templated by the Li_2_Ga (131) plane.

In order to verify whether <110>-oriented single-crystal Li induces directional plating/stripping of the metal, we performed *in situ* 1D-XRD tests of Li|GC-LLZTO-GC|Li and Li|LLZTO-GC|Li cells during first charge/discharge at 0.1 mA cm^−2^ for 10 h. The XRD pattern of an Li|GC-LLZTO-GC|Li symmetrical cell shows that only the intensity of the Li (110) peak changes during the plating/stripping and no other diffraction peaks appear in the process (Fig. 3f). This result proves that the <110>-oriented single-crystal Li metal achieves directional plating and stripping in only the (110) plane. In addition, the *ex situ* SEM images of the LLZTO-GC|Li structure show that the GC interface layer has excellent stability and retains tight contact with both LLZTO and Li metal during the entire plating/stripping process, which leads to a constant overpotential during stable cycling (Fig. S21a). Furthermore, the thickness of the GC layer remains almost constant during cycling, which indicates that the Li ions were mainly deposited on the top of the interface layer. This is confirmed by the regular expansion/contraction of LLZTO-GC|Li structure during Li plating/stripping as indicated by *in situ* SEM imaging (Fig. S21b–S21g and Video S1). Overall, the Li_2_Ga (131) plane on the top of the GC buffer layer has excellent structural stability and maintains its orientation parallel to the interface. The GC|Li interface layer transports Li ions uniformly and efficiently, which causes the Li (110) plane to grow on top of Li_2_Ga (131) so that it is always parallel to the interface and produces highly reversible and directional Li plating/stripping. Because of this, the LLZTO maintains excellent integrity during cycling and ensures the excellent long cycling stability of Li|GC-LLZTO-GC|Li symmetrical cells (Fig. S22a).

In contrast, for the Li|LLZTO-GC|Li asymmetrical battery, the *in situ* 1D-XRD patterns show that the Li plating/stripping in LLZTO|Li occurs simultaneously on the Li (110), (200) and (211) planes because of the polycrystal Li (Fig. 3g). It should be noted that Li plating at the LLZTO|Li interface corresponds to Li stripping at the LLZTO-GC|Li interface and the voids at the LLZTO|Li interface are repaired during plating (Fig. S22b), and therefore there is a quite small and stable overpotential during this process. But Li stripping at the LLZTO|Li interface leads to the formation of Li voids because of the polycrystal Li, and this void formation continues to increase with Li stripping and causes a rapid increase of the overpotential (Fig. 3g). Moreover, the Li filament damages the structure of LLZTO in the LLZTO|Li (Figs S22a and S23), which also leads to the poor cycling stability of an Li|LLZTO-GC|Li asymmetrical battery. Therefore, it is necessary to tune the various crystal orientations of polycrystalline Li metal to <110>-oriented single-crystal Li metal.

### Formation and properties of Li metal with other crystal orientations

**Figure 4.**
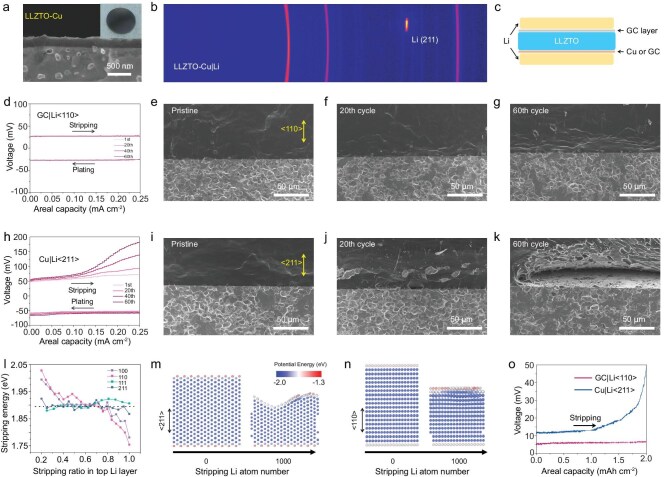
Properties of an Li metal anode with different crystal orientations. (a) Cross-section SEM image and photograph of LLZTO-Cu. (b) XRD pattern of the LLZTO-Cu|Li. (c) Schematic of the assembled Li–Li battery. (d–g) Overpotential curve and the corresponding cross-section SEM images of LLZTO-GC|Li at different cycles at 0.5 mA cm^−2^. (h–k) Overpotential curve and the corresponding cross-section SEM images of LLZTO-Cu|Li at different cycles at 0.5 mA cm^−2^. (l) Stripping energy of common Li crystal planes. (m and n) Dynamic Li stripping behavior of Li (211) and Li (110) crystal planes. (o) Li stripping test of LLZTO-Cu|Li and LLZTO-GC|Li at 0.1 mA cm^−2^ and an areal capacity of 2 mAh cm^−2^.

Whether all single crystal orientations of Li metal can achieve excellent performance remains a question. A <211>-oriented single-crystal Li metal was obtained on a Cu foil after its reaction with Li metal at 300°C (Fig. S24). Therefore, a Cu layer with a thickness of 100–200 nm was sputtered on the surface of a LLZTO ceramic by magnetron sputtering (Fig. 4a) and then reacted with Li metal at 300°C to form Li_x_Cu and obtain the LLZTO-Cu|Li (Fig. S24). XRD patterns show that the Li metal has a strong Li <211> crystal orientation (Fig. 4b). Cross-sectional SEM images indicate that the LLZTO has intimate contact with the Li metal due to the formation of a CuLi_x_ alloy interface (Figs S24 and S25).

Li|GC-LLZTO-Cu|Li and Li|GC-LLZTO-GC|Li batteries were assembled to investigate the Li deposition and stripping at the different interfaces (Fig. 4c). During cycling at a high current density of 0.5 mA cm^−2^ for 0.5 h, the overpotential of the Li|GC-LLZTO-GC|Li cell after stripping during the 1st cycle is 29.1 mV, remains stable during cycling and even slightly decreases to 27.3 mV at the 60th cycle (Fig. 4d). SEM images show that the GC|Li interface has excellent stability and maintains intimate contact during long cycling (Fig. 4e–g). However, the stripping overpotential of the Li|GC-LLZTO-Cu|Li cell gradually increases during cycling. The overpotential after stripping is 72.5 mV during the 1st cycle and increases to 93, 140.1 and 182.9 mV after the 20th, 40th and 60th cycles, respectively (Fig. 4h). Cross-section SEM images show obvious void formation at the interface, and this increases with increasing number of cycles (Fig. 4i–k). These results demonstrate that not all single-crystal orientations of the Li metal are beneficial to the electrochemical performance.

To reveal the intrinsic mechanism of this phenomenon, the thermodynamic properties of Li stripping from several common Li crystal planes (Fig. S26) were calculated by molecular dynamics simulation (Fig. 4l–n; see methods of Li stripping simulation in Methods). It is interesting to note that there are two types of Li metal crystal planes. For (110) and (100) planes, the stripping energy of Li atoms is initially relatively high at the beginning of stripping, and gradually decreases as the number of atoms removed increases. In contrast, the stripping energy for (211) and (111) planes remains almost constant. There are therefore significant differences in the Li stripping thermodynamic properties for different crystal planes, which leads to their quite different Li stripping/plating dynamic behaviors (Fig. 4m and n). For the Li (110) plane, since the stripping energy of the unstripped remaining surface Li atoms is lower than that of a fresh layer (Fig. 4l), the remaining surface Li atoms are preferentially stripped, and the whole interface remains smooth (Fig. 4n, Fig. S27a and Video S2) [28].

In the case of an Li (211) plane, the Li atoms in the inner layer are simultaneously stripped, as well as those in the surface layer due to their similar stripping energy, which leads to the formation of the voids (Fig. 4m, Fig. S27b and Video S3). Accordingly, for polycrystal Li with both (110) and (211) planes parallel to the surface, Li atoms prefer to strip from (211) due to the lower initial stripping energy and form voids (Fig. S28 and Video S4), resulting in an uneven interface and poor physical contact with the interface. This uneven surface produces a non-uniform distribution of localized electrical field, which leads to the preferential deposition of Li ions on an ‘Li tip’ with a higher charge density during the next plating cycle, resulting in the generation of Li dendrites. Therefore, the different Li stripping thermodynamic properties for various crystal planes is the basic reason for the interface deterioration, which induces Li dendrite formation.

A large area test of the Li stripping capacity of LLZTO-Cu|Li and LLZTO-GC|Li was also performed at a current density of 0.1 mA cm^−2^ and an areal capacity of 2 mAh cm^−2^ to confirm the interface stability contributed by the <110>-oriented single-crystal Li metal (Fig. 4o). The initial stripping overpotentials of LLZTO-Cu|Li and LLZTO-GC|Li were 11.2 and 5.6 mV, respectively, with the latter increasing only to 6.5 mV after 2 mAh cm^−2^ Li stripping and 7.8 mV after 2.35 mAh cm^−2^ Li stripping (Fig. S29a). In sharp contrast, the overpotential of LLZTO-Cu|Li increases to 49.3 mV after 2 mAh cm^−2^ Li stripping and sharply increases to 1304.6 mV after 2.3 mAh cm^−2^ Li stripping (Fig. S29a). In addition, the interface resistance of LLZTO-GC|Li only changes from 1.5 to 1.7 Ω cm^2^ after 2.35 mAh cm^−2^ Li stripping (Fig. S29b), whereas the interface resistance of LLZTO-Cu|Li increases from 5.5 to 221.0 Ω cm^2^, which means a very severe interface degradation (Fig. S29c and S29d). These results also confirm that the Li atoms in the (110) plane tend to be peeled off layer-by-layer during the stripping process to maintain a smooth surface, which ensures excellent physical contact and a stable overpotential, while the Li (211) plane is more likely to strip perpendicular to the plane to form voids, which results in interface deterioration and Li dendrite generation during long cycling.

### Outstanding performance of LLZTO-GC|Li-based batteries

**Figure 5.**
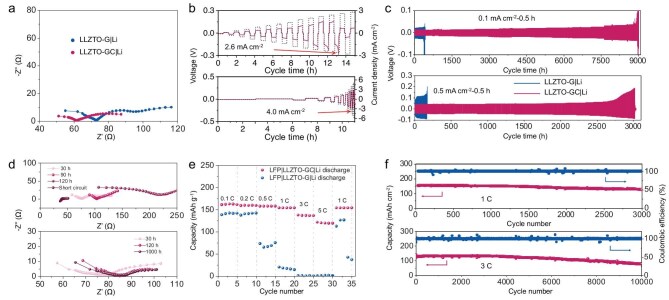
Characterizations of LLZTO-GC|Li-based batteries. (a) EIS plots of Li|GC-LLZTO-GC|Li and Li|G-LLZTO-G|Li symmetrical batteries. (b) CCD test with constant charge/discharge time (0.5 h) and constant capacity (0.3 mAh cm^−2^) of Li|GC-LLZTO-GC|Li. (c) Cycling performance of Li|GC-LLZTO-GC|Li and Li|G-LLZTO-G|Li at 0.1 and 0.5 mA cm^−2^. (d) *Ex situ* EIS plots of Li|GC-LLZTO-GC|Li and Li|G-LLZTO-G|Li symmetrical batteries after different cycles at 0.5 mA cm^−2^. (e) Rate performance of LFP|LLZTO-GC|Li and LFP|LLZTO-G|Li batteries with cathode mass loading of 2 mg cm^−2^ and an areal capacity of 0.34 mAh cm^−2^. (f) Cycling performance of LFP|LLZTO-GC|Li battery at 1 and 3 C with a cathode mass loading of 2 mg cm^−2^. All the electrochemical performances were tested at 25°C. The current density of 1 C is 170 mA g^−1^.

Further electrochemical tests were performed to demonstrate the excellent performance of the perfect Li <110>-oriented LLZTO-GC|Li structure. The interface impedance of LLZTO-GC|Li is only 0.76 Ω cm^2^, which is much smaller than that of LLZTO-G|Li (1.3 Ω cm^2^) and Li|LLZTO|Li cells (220 Ω cm^2^) (Fig. 5a and Fig. S30a). The greater bonding force of GC with both Li metal and LLZTO results in smaller interface impedance (Fig. S30b). As a result, the Li|GC-LLZTO-GC|Li symmetrical battery exhibits a quite high critical current density (CCD) of 2.6 mA cm^−2^ with a constant charge/discharge time of 0.5 h, and 4.0 mA cm^−2^ with a constant capacity of 0.3 mAh cm^−2^ (Fig. 5b). The Li|LLZTO|Li symmetrical battery without the modified layer shows a short-circuit phenomenon within a few minutes at a current density of 0.1 mA cm^−2^ (Fig. S30c). Although the Li|G-LLZTO-G|Li cell has a low interface impedance and can maintain a low overpotential during the initial cycle, its cycle life is far inferior to that of Li|GC-LLZTO-GC|Li. Specifically, the cycle life of the Li|GC-LLZTO-GC|Li cell is 9000 h at 0.1 mA cm^−2^ and 3000 h at 0.5 mA cm^−2^, while the cycle life of Li|G-LLZTO-G|Li is only 400 h at 0.1 mA cm^−2^ and 160 h at 0.5 mA cm^−2^, respectively (Fig. 5c), and its CCD (1.0 mA cm^−2^) is much lower than that of Li|GC-LLZTO-GC|Li (Fig. S30d). *Ex situ* EIS plots were performed to monitor the interface evolution during cycling, as shown in Fig. 5d. The impedance of Li|G-LLZTO-G|Li increases significantly within hundreds of cycles. In sharp contrast, the impedance of Li|GC-LLZTO-GC|Li only increases slightly during initial cycles and keeps constant basically in the subsequent thousands of cycles. In order to verify the practical prospects of this interface, we also cycled the Li|GC-LLZTO-GC|Li symmetrical battery at a high current density. Its cycle life reaches over 450 h at 1 mA cm^−2^ (Fig. S30e). The cycling performance of the Li|GC-LLZTO-GC|Li symmetrical battery shows a significant improvement compared to those of previous reports (Fig. S31) [5,17–19,29–37].

To further confirm that <110>-oriented single-crystal Li is the key reason for improving the cyclability of Li metal, we designed a control experiment to produce the same interfacial layer but different crystal orientations of Li metal. We found that the GC layer can react with molten Li at different temperatures, such as 200°C, 250°C and 300°C to form almost the same interfacial layer but construct Li metal with quite different crystal orientations. The XPS of the GC layer after reaction with Li metal at 200°C, 250°C and 300°C shows that the GC interfacial layer has the same interfacial compositions, mainly including Li_2_Ga and LiGaO_2_ (Fig. S32), while the Li metal presents quite different crystal orientation, as shown in 2D/XRD images (Fig. S33). Specifically, the Li metal with (110), (200) and (211) crystal planes is formed on the GC layer after reaction with Li metal at 200°C, while the Li (200) crystal plane disappears when the reaction temperature increases to 250°C. Only the single-crystal Li (110) plane is retained with the reaction temperature increasing to 300°C. It is noted from Fig. S34 that the Li|GC-LLZTO-GC|Li symmetrical cells using Li metal with Li (110), Li(200) and Li(221) crystal planes obtained at 200°C and 250°C present rapid short circuits, which present much poorer cycling stability than the Li|GC-LLZTO-GC|Li symmetrical cells using single-crystal Li with only the (110) plane obtained at 300°C. The above results clearly present that the application of the same interfacial layer but quite different crystal orientation of Li presents entirely different cycling stability, confirming that the <110>-oriented single-crystal Li is the key and decisive reason for improving cyclability of Li metal.

We further paired the LLZTO-GC|Li structure with an LFP cathode to assemble the ASLMBs. The interface layer used on the cathode side is succinonitrile (SN) electrolyte (Fig. S35a). The assembled LFP|LLZTO-GC|Li battery with a cathode mass loading of 2 mg cm^−2^ and an areal capacity of 0.34 mAh cm^−2^ delivers a high specific capacity of 120 mAh g^−1^ at 5 C (Fig. 5e and Fig. S35b). When the current density returns to 1 C, the capacity also recovers to 154 mAh g^−1^. After the shock of high current density, the battery still can stably cycle 3000 times with a capacity retention of 83% (Fig. 5f). In sharp comparison, the capacity of the LFP|LLZTO-G|Li battery is almost zero at 5 C (Fig. 5e). When the current density returns to 1 C again, the battery immediately experiences an overcharge phenomenon caused by short-circuit (Fig. S35c). This is due to the destructive damage of LLZTO caused by Li dendrite growth and visible cracks are formed (Fig. S35d), which eventually leads to the short-circuit of the battery. At a current density of 3 C, the LFP|LLZTO-GC|Li battery exhibits an extraordinarily high capacity retention of 92% after 6000 cycles and 62% after 10 000 cycles (Fig. 5f). We also assembled the LFP|LLZTO-GC|Li battery with a high cathode mass loading of 7 mg cm^−2^ and an areal capacity of 1.22 mAh cm^−2^, which also presents stable cycling performance (Fig. S36). The coulombic efficiency of the LFP|LLZTO-GC|Li battery is maintained at about 100% during the whole cycling. Therefore, the LFP|LLZTO-GC|Li full batteries present excellent cycling stability and markedly outperform previously reported LLZTO-based systems (Table S2) owing to the precise regulation of polycrystal Li metal to <110>-oriented single-crystal Li, which fundamentally solves the Li dendrite formation and interface degradation in ASLMBs.

## CONCLUSION

We have established that the multi-directional Li stripping caused by the different orientations of the polycrystal Li is the basic cause for Li dendrite generation and interface degradation in ASLMBs. The preferred <110>-oriented single-crystal Li has been successfully obtained by using a lattice-matching template of a Li_2_Ga (131) interface in the melting/recrystallization of the molten polycrystal Li. Li atoms on the Li (110) crystal plane undergo layer-by-layer stripping, which avoids the formation of voids at the interface with LLZTO. This fundamentally ensures excellent contact between the Li metal anode and the LLZTO during long cycling, producing ultralong cycling lives both for the experimental symmetrical and practical full batteries. Typically, LFP-matched ASLMBs using Li metal with <110> crystal orientations exhibit an ultra-high-capacity retention of 92% after 6000 cycles at 3 C and 25°C. The fine regulation of the preferred crystal orientation of Li metal has shown great potential as a final solution to overcome the huge challenges of dendrite formation and interface degradation and may greatly speed up the industrial use of ASLMBs.

## Supplementary Material

nwaf540_Supplemental_Files
