# Supplementary material for: Single-crystal orientation lithium for ultra-stable all-solid-state batteries
Source: Natl Sci Rev. 2025 Dec 1;13(2):nwaf540. doi: 10.1093/nsr/nwaf540 (PMC12839520; doi:10.1093/nsr/nwaf540)
Supplement: nwaf540_Supplemental_Files [file nwaf540_supplemental_files.zip › Supplementing data.pdf]

Supplementary Data for

Single-crystal orientation lithium for ultra-stable all-solid-state batteries

Qidong Li[1†], Likun Chen[1†], Junyu Jiao[2†], Yang Zhao[1†], Suting Weng[3], Jun Zhao[4], Jiabin Ma[1], Yuhang Li[1], Genming Lai[2], Shichao Wu[5], Xufei An[1], Ke Yang[1], Jie Biao[1], Xing Cheng[1], Kai Shi[1], Jiaxin Zheng[2*], Xuefeng Wang[3], Yongfu Tang[4], Ming Liu[1], Lele Peng[1], Wei Lv[1], Jun Lu[6*], Feiyu Kang[1*], Quan-Hong Yang[5*], Yan-Bing He[1*]

[1] Shenzhen All-Solid-State Lithium Battery Electrolyte Engineering Research Center, Institute of Materials Research (IMR), Tsinghua Shenzhen International Graduate School, Tsinghua University, Shenzhen 518055, China.

[2] School of Advanced Materials, Peking University, Shenzhen Graduate School, Shenzhen 518055, China.

[3] Beijing Key Laboratory for New Energy Materials and Devices, Institute of Physics, Chinese Academy of Sciences, Beijing 100190, China.

[4] Clean Nano Energy Center, State Key Laboratory of Metastable Materials Science and Technology, Yanshan University, Qinhuangdao 066004, China.

[5] Nanoyang Group, Tianjin Key Laboratory of Advanced Carbon and Electrochemical Energy Storage, State Key Laboratory of Chemical Engineering, School of Chemical Engineering and Technology, Tianjin University, Tianjin 300072, China.

[6] College of Chemical and Biological Engineering, Zhejiang University, Hangzhou 310027, China.

† These authors contributed equally to this work.

[*]**Corresponding author**. E-mail: zhengjx@pkusz.edu.cn, junzoelu@zju.edu.cn, fykang@sz.tsinghua.edu.cn, qhyangcn@tju.edu.cn, he.yanbing@sz.tsinghua.edu.cn

Methods

Preparation of LLZTO

$Li_{6.4}La_3Zr_{1.4}Ta_{0.6}O_{12}$ (LLZTO) pellets were sintered by spark plasma sintering. 1.1 g LLZTO ceramic powder was placed in a graphite mold with a diameter of 10 mm, which was sintered at 1100°C for 10 min with a pressure of 50 MPa. The sintered LLZTO ceramic plate was polished by sandpaper into a smooth wafer with a thickness of 500 μm.

Preparation of LLZTO-Cu and LLZTO-GC

A Cu film was magnetron-sputtered onto the surface of a LLZTO ceramic wafer to form LLZTO-Cu. The sputtering was conducted in an Ar atmosphere, the pressure was 2 Pa, the power was 80 W, the distance from the Cu target to LLZTO wafers was 6 cm, and the sputtering time was 10 min. LLZTO-GC was prepared as follows. A Ga-based alloy (Ga: 72.16 At%, In: 18.59 At%, Sn: 9.25 At%) was blended with 5 wt% N-doped carbon nanotubes (CNTs) by a vortex oscillator. The blended fluid was painted onto the surface of a LLZTO ceramic wafer in air to obtain LLZTO-GC. The CNTs with a N content of 3 wt% were purchased from Macklin. Transmission electron microscopy (TEM) images of the N-doped CNTs are shown in Fig. S1A and 1B.

Reaction of different interfaces with melting Li metal

The oxide layer on a piece of Li metal was removed with a knife, and it was then rolled into sheets with a thickness of ~300 μm. The sheets were cut into round Li pieces with a hole punch of 8 or 10 mm and were stacked with different interface materials and placed on a heating plate in a glove box at a temperature of 300°C for 2 min and were then cooled naturally to room temperature. The Li-Li batteries were prepared with the same procedure using LLZTO ceramic wafers decorated with different interface materials on both sides beforehand.

Preparation of electrodes

Electrode slurries were prepared by mixing the $LiFePO_4$ active material, carbon black (Super P, Alfa Aesar) and binder (polyvinylidene fluoride, MTI) in weight ratios of 8:1:1 in a N-methyl-2-pyrrolidone (Sigma-Aldrich) solution. Electrode sheets were prepared by coating the slurry onto Al foils with a doctor blade and were then dried in a vacuum oven at 80°C for 12 h. The mass loading was 2-7.2 mg cm^{-2}. The succinonitrile electrolyte used on the cathode side was prepared by mixing 2.0 g of succinonitrile and 0.4 g of LiTFSI and stirring at 60°C for 2 hours to ensure complete dissolution of LiTFSI.

Electrochemical Characterizations

The CR2032 coin cells were all assembled in an argon-filled glovebox (O_2 < 0.1 ppm, H_2O < 0.1 ppm). The electrochemical impedance spectroscopy (EIS) is performed by an electrochemical station (VMP3, Bio-Logic Science Instruments) from 7 MHz to 0.1 Hz (a.c. oscillation = 10 mV). The galvanostatic charge/discharge tests of cells were performed on the LAND CT2001A battery test system.

Characterizations

1D/2D X-ray diffraction (XRD) and operando XRD were obtained using a NEW SmartLab 9KW X-ray diffractometer with Cu Kα radiation (λ = 1.5418 Å) at room temperature. The scanning mode was θ/2θ mode from 10 °–80 ° with a scan rate of 7 ° min^{-1}. The morphology and element distribution of the LLZTO sheet and interface layer were analyzed by field emission scanning electron microscope (FE-SEM, HITACHI S4800, Japan) and its associated energy spectrum system. The phase composition of the GC interface layer was analyzed by TEM (FEI Tecnai G2 F30). X-ray photoelectron spectroscopy (XPS) (PHI5802) was used to analyze the valence states of elements before and after the GC layer reacted with Li metal. High-resolution micrographs of LLZTO-GC|Li were recorded on a cryo-TEM. The LLZTO-GC|Li pellet was first transferred under cryogenic conditions to an ultramicrotome (Leica EM UC7/FC7) and sliced into ultrathin pieces with a fresh LLZTO-GC|Li interface. The collected pieces were loaded onto a TEM grid, which was then immersed in liquid nitrogen and transferred to a glovebox without exposure to air. The TEM samples were loaded onto the cooling holder inside the glovebox and

transferred to the TEM system under continuously flowing argon gas. Cryo-images were acquired using the microscope (JEOL JEM F200) at 178ºC and 200 kV. The in-situ SEM experiment was performed in a FIB-SEM system (Helios G4 CX, Thermo Fisher Scientific).

Stripping simulation

The stripping simulations of Li panels with different crystal orientations were carried out by molecular dynamic simulation using LAMMPS [1]. A Li surface potential (Li-SP) [2] was used to drive LAMMPS and calculate the energy and force for every Li atom. Li-SP was constructed by a deep neural network [3] and trained using datasets based on ab initio calculation. Li-SP can obtain accurate ab initio calculations for both bulk and surface Li. The mean absolute errors on energy are 0.85 meV/atom and 1.21 meV/atom for bulk and surface tests, respectively [2].

The potential energy of each Li atom was calculated, and the Li atom with the highest potential energy was then stripped. The new Li surface structure with stripped Li atoms was fully relaxed to reach its potential energy minimization. The above process was repeated until the number of dropped Li atoms reached a set value. The visualizations of the atomic structures were performed with the OVITO [4] and VESTA [5] programs.

The stripping energy is defined as:

$$E_s = \frac{E_{Li}(m-n) - E_{Li}(m) + nE_{atom}}{n}$$

where E_s is the stripping energy, n is the number of stripped Li atoms and m is the total number of Li atoms in the Li plane before Li atom stripping. $E_{Li}(m)$ and $E_{Li}(m-n)$ are respectively the total Li potential energies before and after n Li atoms have been stripped. E_{atom} is the energy of an isolated Li atom (~2.4 meV), which is negligible compared with the stripping energy. The higher the E_s, the harder the Li atom stripping.

Simulation of Li growth on Li_2Ga

In the Li growth simulation, an ab initio molecular dynamic simulation (AIMD) was carried out by the Vienna ab initio simulation package (VASP) [6, 7] based on density functional theory (DFT) to study Li atom behavior on the Li_2Ga surfaces. The generalized gradient approximation (GGA) with a parametrized exchange-correlation function according to Perdew Burke and Ernzerhof (PBE) was used during the calculation [8]. The valence electron wave functions were expanded in the plane wave basis sets, and the projector augmented wave (PAW) method was used to describe the core-electron interactions [9]. The Brillouin zone samplings were performed using a 1 × 1 × 1 k-point grid in the Monkhorst-Pack scheme for the AIMD simulation.

Li_2Ga with an exposed (131) surface and Li with an exposed (100) surface were constructed and these two surfaces were merged to construct the Li_2Ga|Li interface. The bottom two layers of the structures were fitted during the AIMD simulation to produce a bulk environment. The AIMD was first carried out at 700 K in the NVT ensemble to melt the Li metal, and the temperature was then gradually reduced to 50 K.

To get a more accurate result, a larger 2×2×1 cell was built based on the Li metal that was generated on the Li_2Ga <131> surface. Molecular dynamic simulation driven by the Li-SP potential was then carried out in LAMMPS [1] to simulate the Li growth, which was carried out in the NVT ensemble with the bottom layer of Li fixed to provide a growth seed. The bottom Li layer is exactly the same as the final Li layer in the Li_2Ga in the above AIMD simulation. The temperature was increased from 300 K to 700 K to melt the Li, and then gradually decreased to 50 K to simulate Li crystallization.

Supplementary Figures

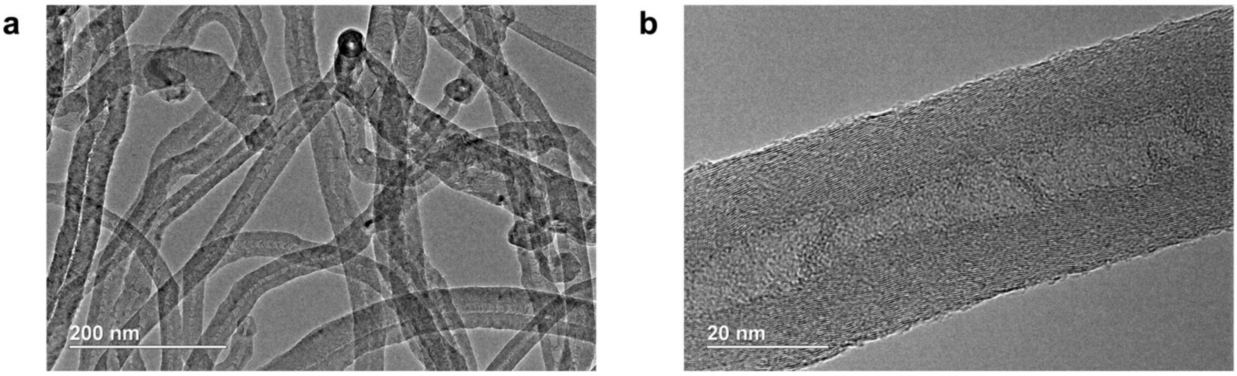

Figure S1. (a, b) TEM images of the N-doped CNTs.

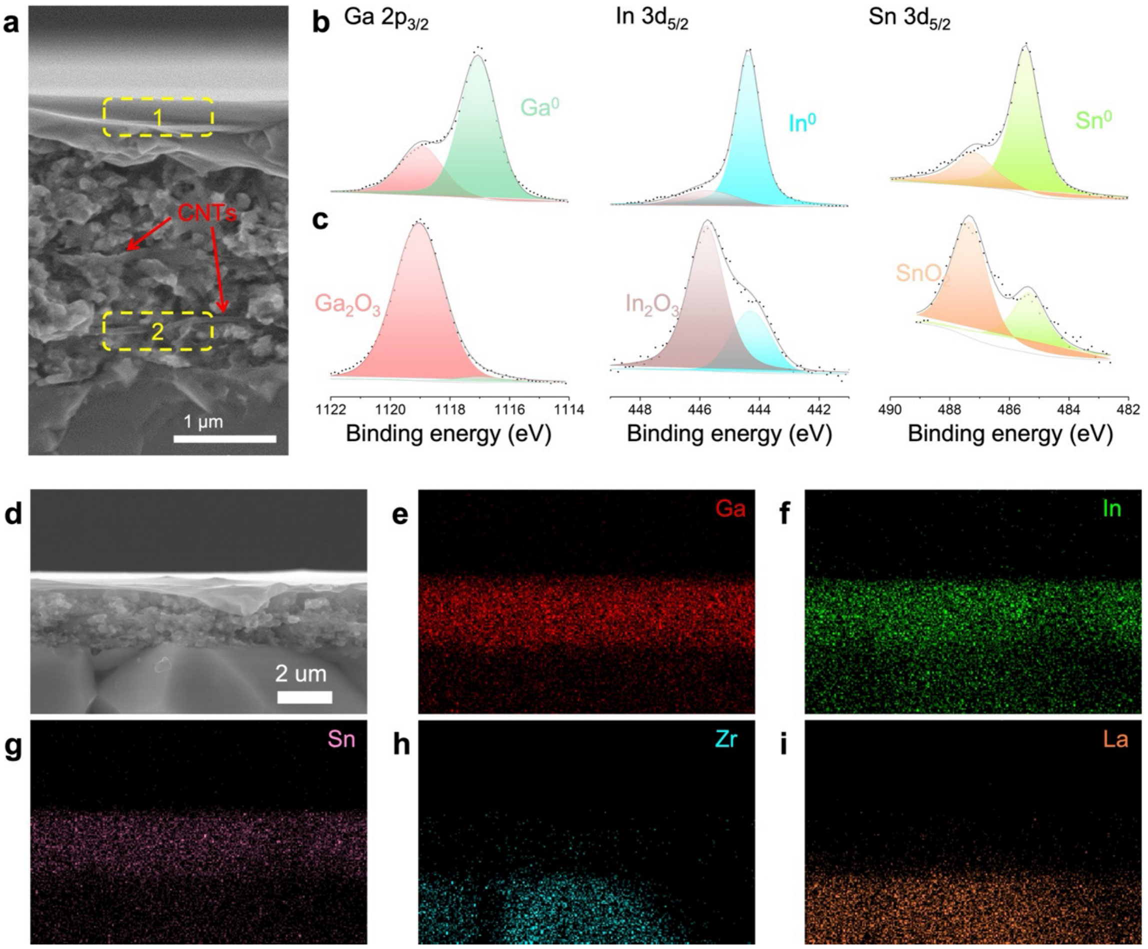

Figure S2. (a) Cross-section SEM image of the prepared LLZTO-GC. (b-c) XPS analysis of the layer GC interface at (b) the top and (c) bottom layer. (d-i) EDS maps of the GC interface layer.

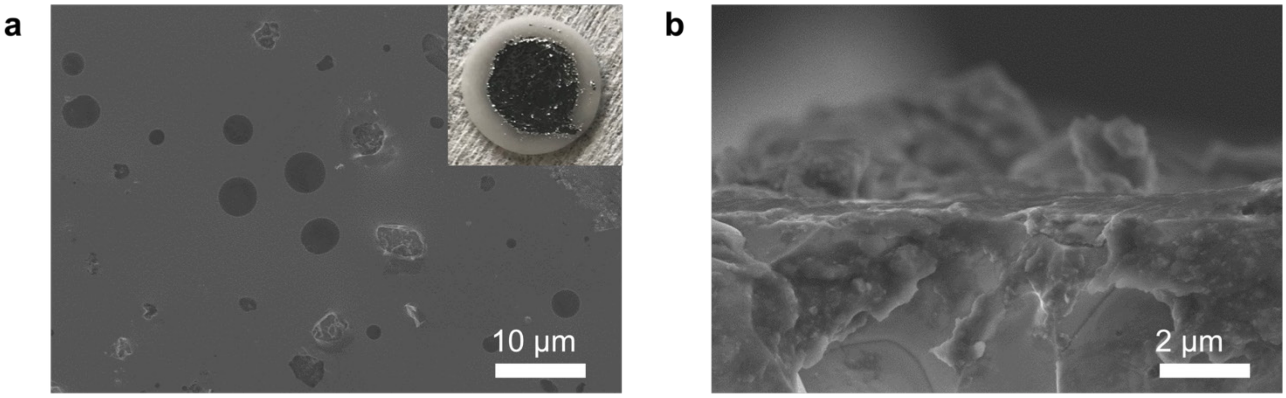

Figure S3. (a) SEM image and optical photograph of the prepared LLZTO-G structure. (b) Cross-section SEM image of the prepared LLZTO-G.

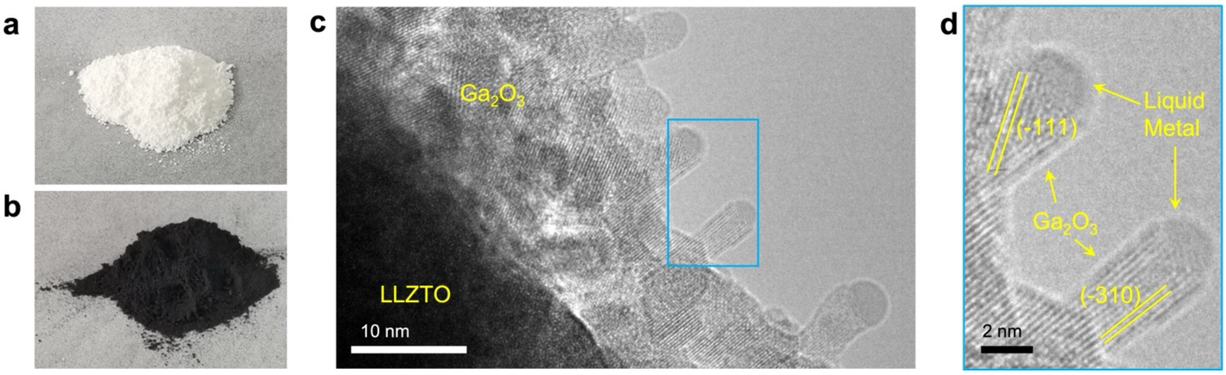

Figure S4. (a) Optical image of the original LLZTO powder and (b) LLZTO powder after grinding with GBA in air (LLZTO-G). (c-d) TEM images of LLZTO-G.

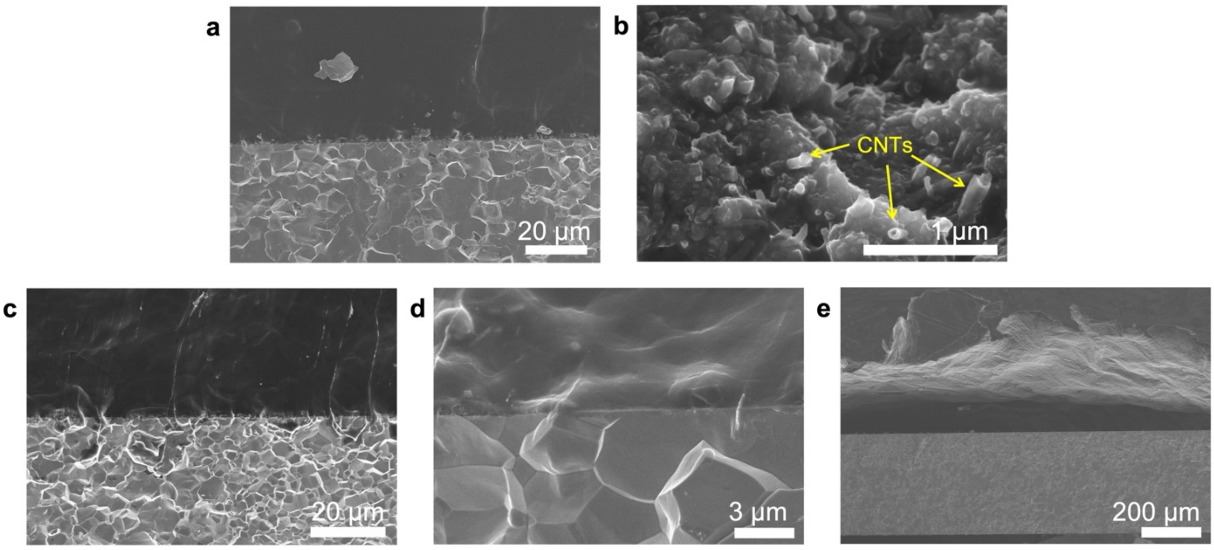

Figure S5. (a, b) Cross-section SEM images of LLZTO-GC|Li. (c-d) Cross-section SEM images of LLZTO-G|Li. (e) Cross-section SEM image of LLZTO|Li.

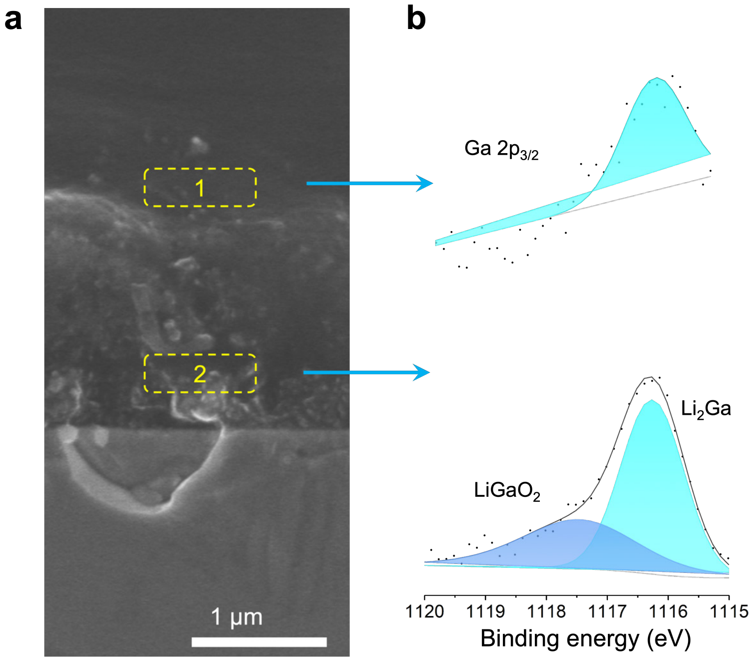

Figure S6. (a) Cross-section SEM images of LLZTO-GC|Li and (b) corresponding XPS analysis of LLZTO-GC|Li at the top and bottom layer.

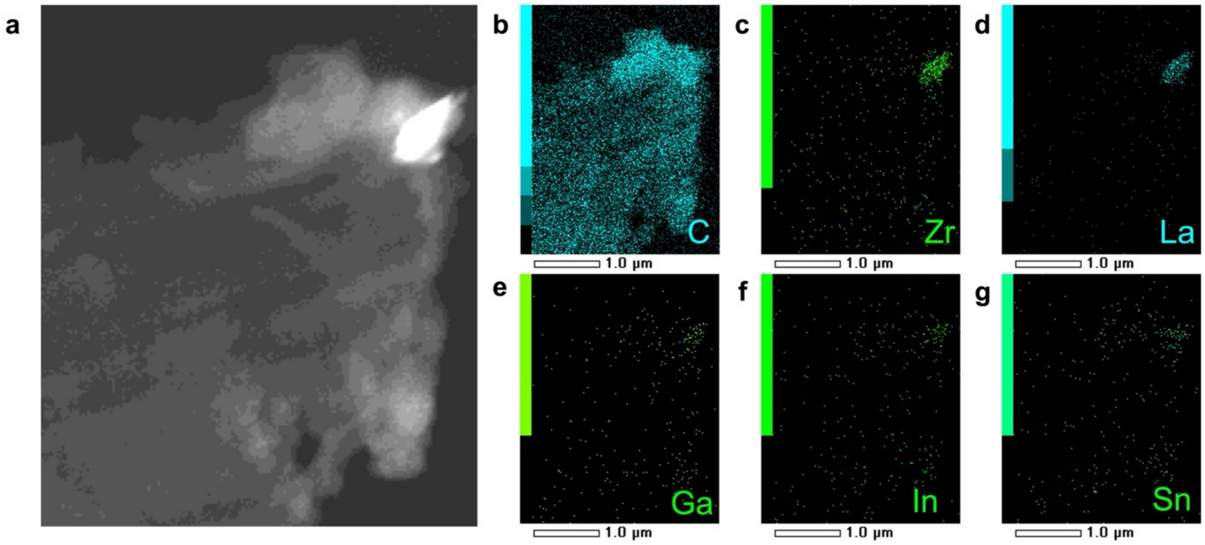

Figure S7. (a-g) The TEM image and EDS maps of LLZTO-GC|Li acquired by cryo-TEM.

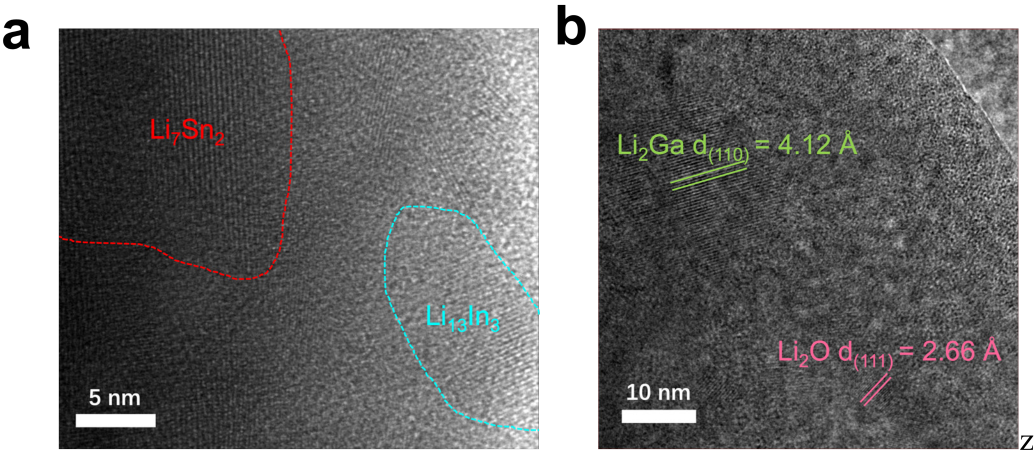

z

Figure S8. (a-b) Cryo-HRTEM images of LLZTO-GC|Li.

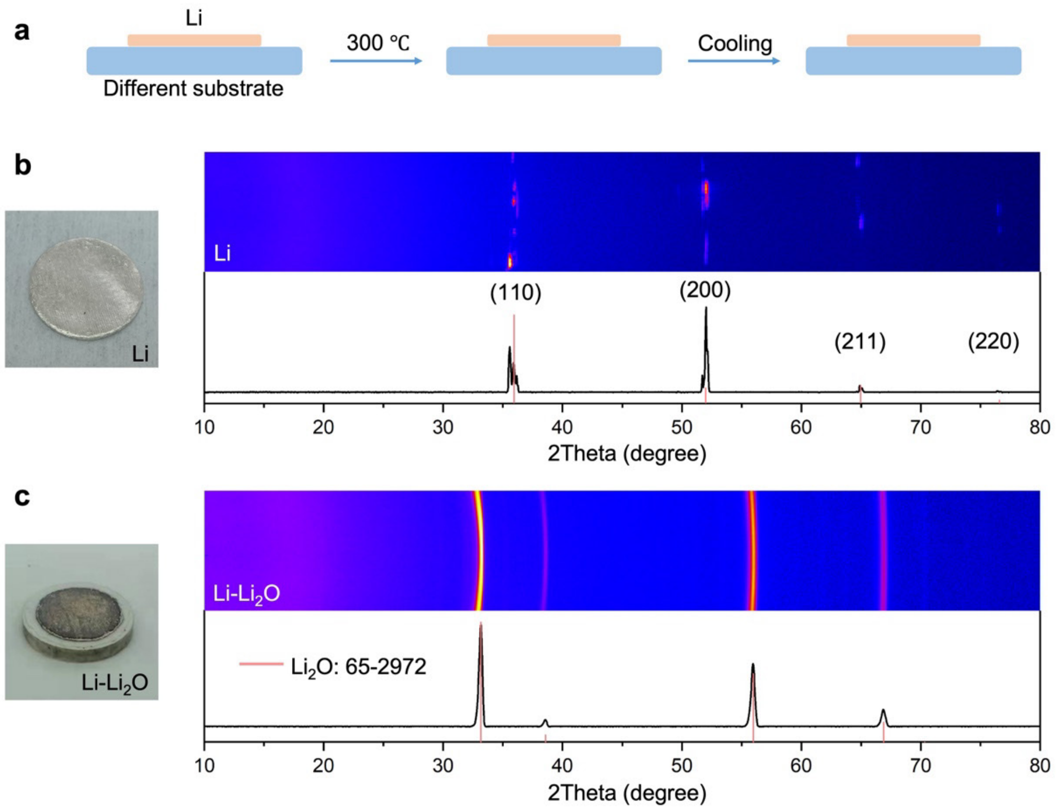

Figure S9. (a) Schematic of melting/crystallization of Li metal on different substrates at 300°C. (b, c) 2D-XRD and corresponding 1D-XRD for the texture of (b) the original Li metal plate and (c) a crystallized Li metal plate on a Li_2O substrate. It should be noted that the discontinuous Debye–Scherrer rings in the 2D-XRD pattern of the original Li metal plate indicate its partial texture, which is attributed to the mechanical rolling process during its manufacturing [10].

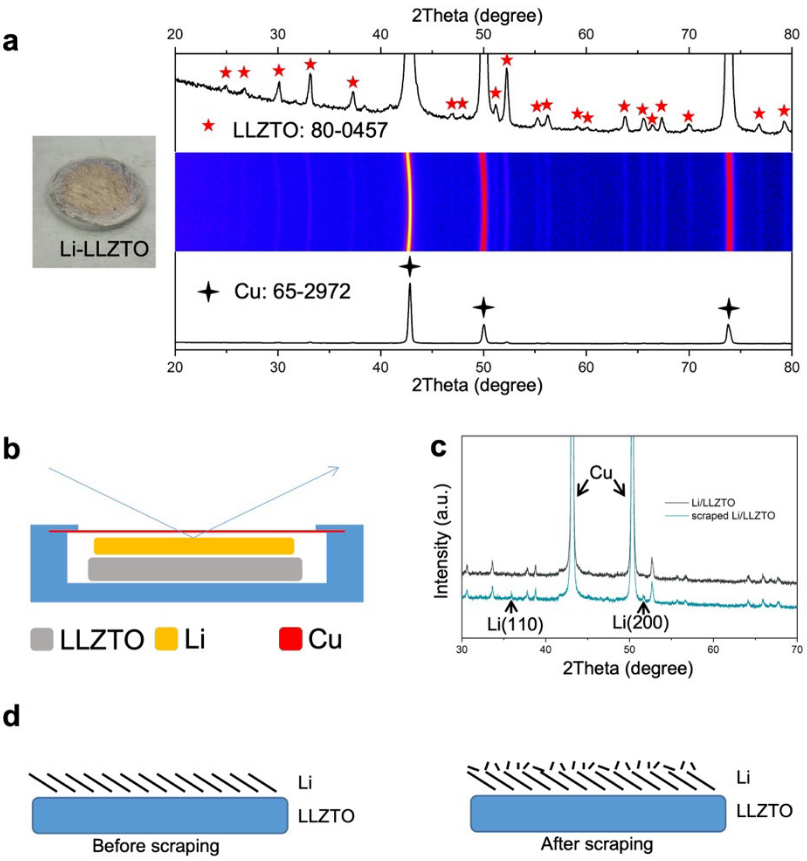

Figure S10. (a) 2D-XRD and corresponding 1D-XRD of Li-LLZTO prepared by recrystallization of molten Li metal on LLZTO after melting at 300°C. (b) Structure schematic of the XRD test mold. (c) 1D-XRD of Li-LLZTO structure before and after scraping. (d) Schematic of Li-LLZTO before and after scraping. In order to avoid contact with air, an XRD mold was used to encapsulate samples. Since copper foil was used in the mold, a copper diffraction signal will appear in the 1D/2D-XRD tests. In order to verify the single-crystal nature of Li formed on the first type of interface, we scraped the Li surface with tweezers to destroy the single-crystal structure of the surface (d). 1D-XRD patterns of Li-LLZTO after scraping (c) clearly show the Li (110) and (200) diffraction peaks, which confirms that the recrystallized molten Li metal on LLZTO tends to form a single crystal with a random orientation due to it having no interfacial reaction with the Li metal.

Figs. S10 and S11 show that there are two main types of interfaces depending on whether the interface reacts with Li at high temperatures. The first interface such as pure LLZTO and Li_2O does not react with Li metal. The Li melting/recrystallization process is close to an ideal uniform nucleation process. The recrystallized Li anode tends to form a single crystal with a random orientation, whose diffraction signal is hardly detected by the 1D/2D-XRD. The second type of interface such as Ga reacts with Li at high temperature. In the Li recrystallization process, the reaction product of Li_2Ga alloy at the interface provides two-dimensional crystal nuclei for molten Li, which can induce the recrystallization of Li metal.

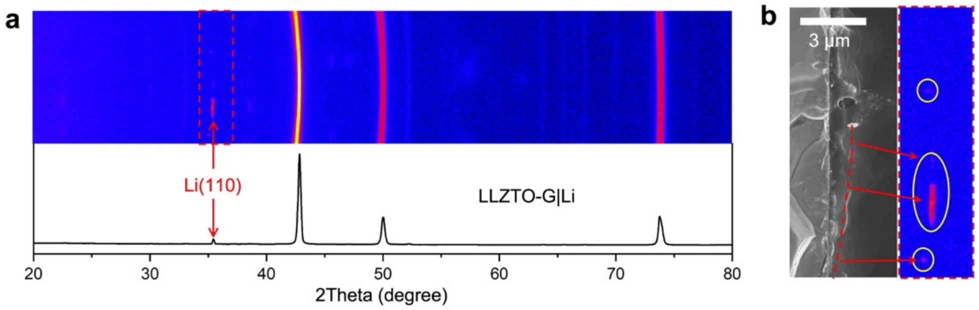

Figure S11. (a) 2D-XRD and corresponding 1D-XRD of the LLZTO-G|Li. (b). 2D-XRD signal of the LLZTO-G|Li with an uneven G layer.

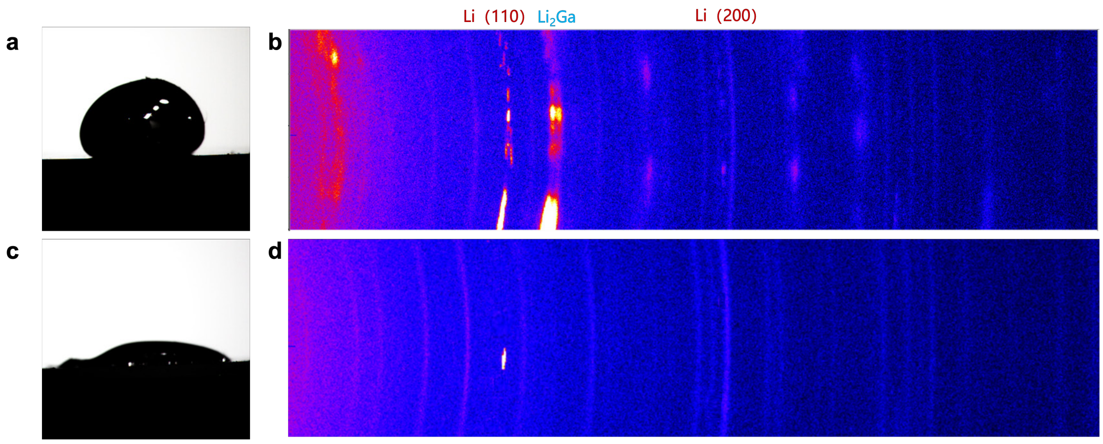

Figure S12. (a) Optical image of the contact angles and (b) 2D-XRD pattern of LLZTO-Ga/CNT|Li after reaction at 300°C. (c) Optical image of the contact angles and (d) 2D-XRD pattern of LLZTO-GC|Li after reaction at 300°C.

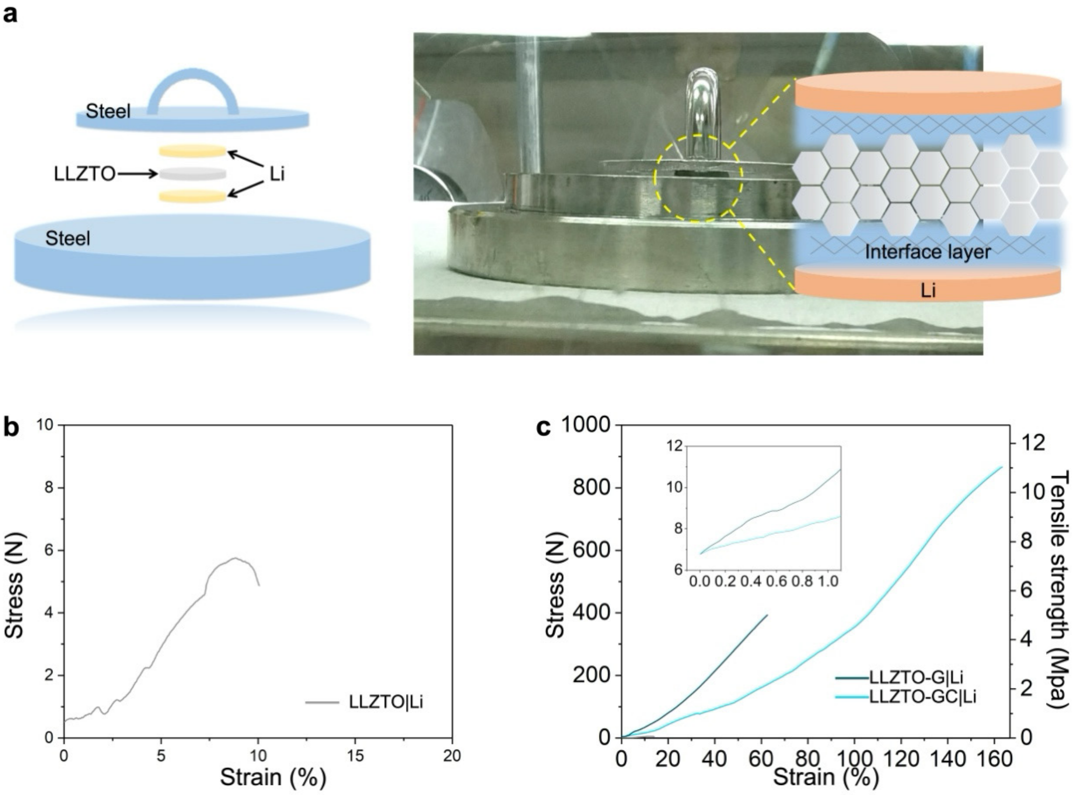

Figure S13. (a) Schematic of the mechanical test. The GC layer was constructed between the Li metal disk and the steel substrate to act as a welding agent. During the heating process, the Li metal reacted with the GC layer to form an alloy, thereby forming a welding effect between the steel substrate and Li metal disk. The resulting intimate contact ensures that the Li metal disk remains firmly attached to the steel substrate without delamination during the tensile test. (b) Stress-strain curves for Li|LLZTO|Li. (c) Stress-strain curves for Li|G-LLZTO-G|Li and Li|GC-LLZTO-GC|Li. LLZTO-GC|Li has a bonding force of 863 N, which is much larger than that of LLZTO-G|Li (393 N) and LLZTO|Li (6 N). The figure also shows that the elastic modulus of LLZTO-GC|Li with a perfect Li<110> crystal orientation is almost half of that of the LLZTO-G|Li with only some Li<110> crystal orientation (Fig. S13c inset). This means that, under the same deformation (or the same amount of Li deposition) condition, the internal stress generated by the LLZTO-GC|Li sample is smaller than that of LLZTO-G|Li, which is critical for the protection of the LLZTO during long cycling.

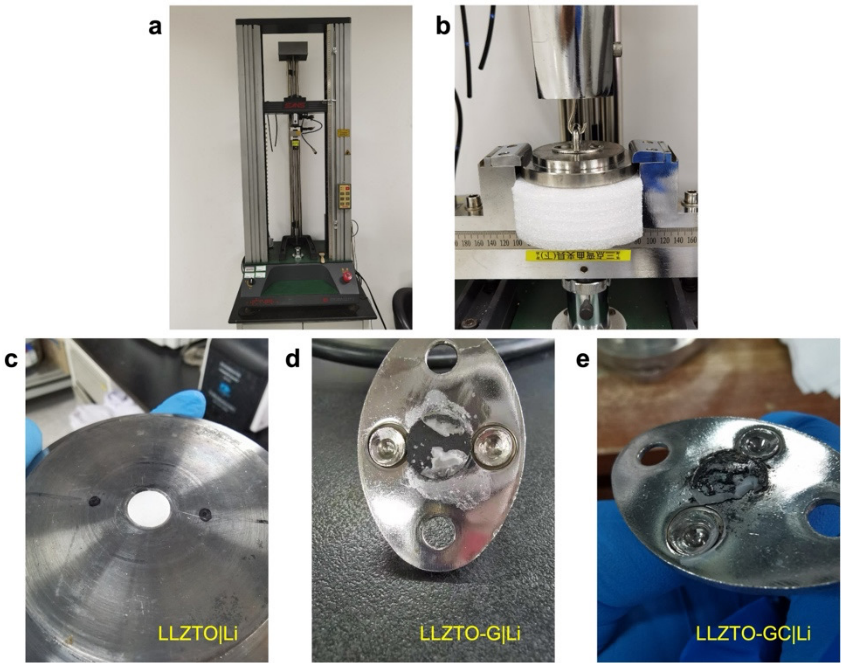

Figure S14. (a-b) Test instrument for the mechanical test. (c-e) Optical photographs of each sample after mechanical testing.

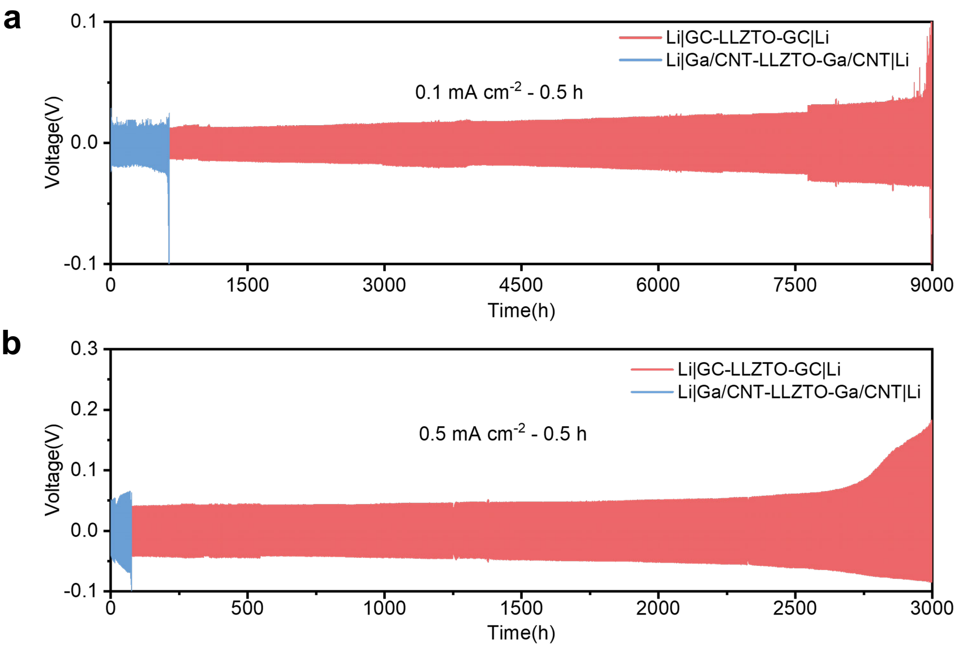

Figure S15. Cycling performance of Li|GC-LLZTO-GC|Li and Li|Ga/CNT-LLZTO-Ga/CNT|Li symmetric batteries under (a) 0.1 mA cm^{-2} and (b) 0.5 mA cm^{-2}.

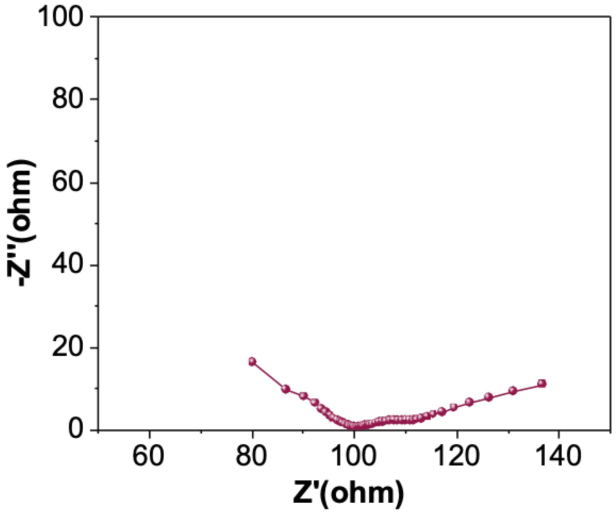

Figure S16. EIS plot of Li|GC-LLZTO-GC|Li symmetric batteries at 0.1 mA cm^{-2} with a specific capacity of 1 mAh cm^{-2} after 2400 h cycling.

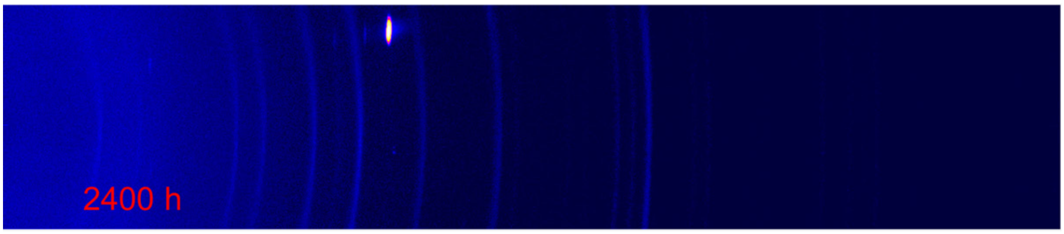

Figure S17. 2D-XRD of LLZTO-GC|Li after 2400 h cycling.

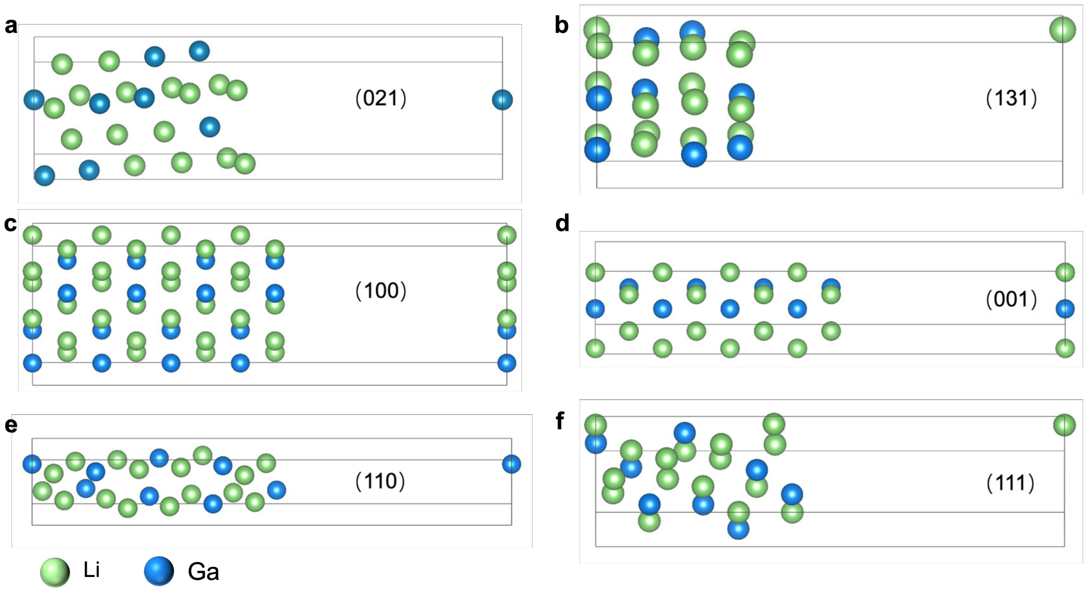

Figure S18. The slab configurations of the main crystal planes of Li_2Ga including (a) (021), (b) (131), (c) (100), (d) (001), (e) (110) and (f) (111).

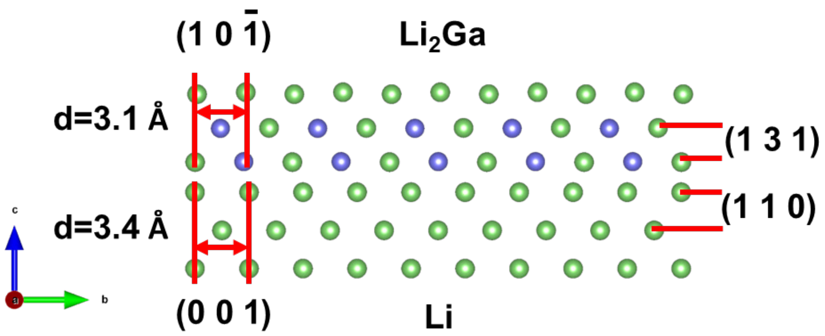

Figure S19. Lattice mismatch between the Li_2Ga (131) and Li (110).

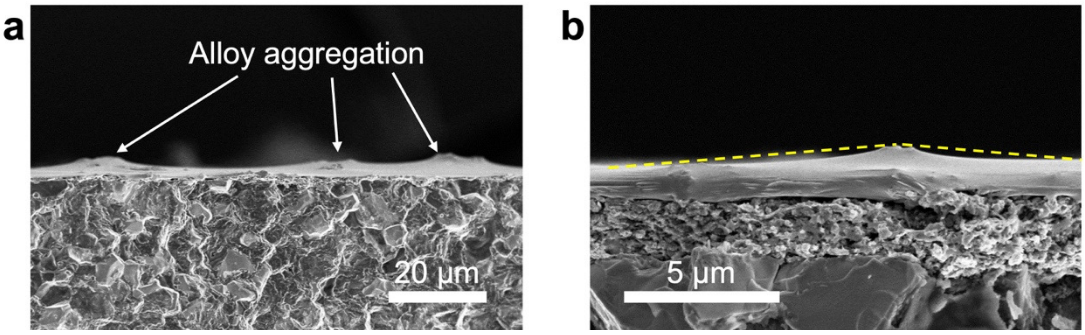

Figure S20. (a, b) Cross-section SEM images of LLZTO-GC with excess GBA and the aggregation of GBA leading to the unevenness of the GC layer.

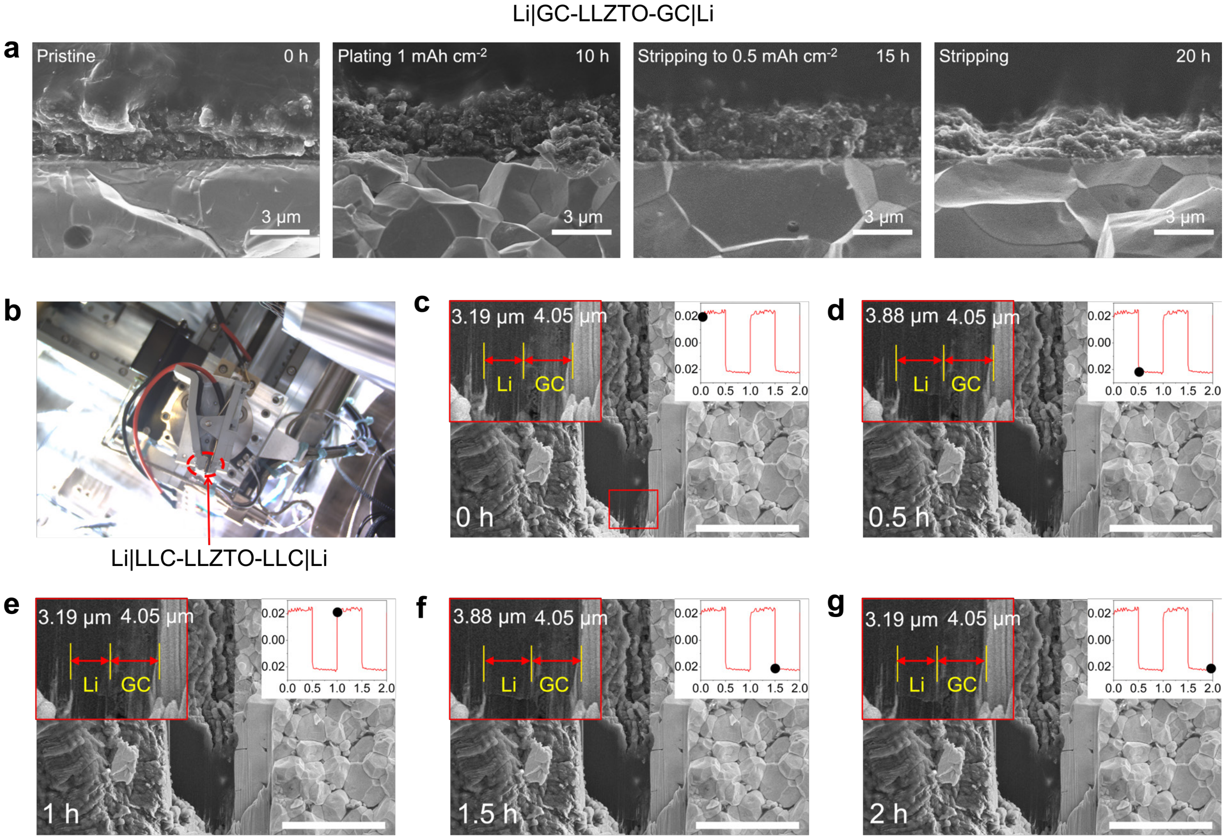

Figure S21. (a) Ex-situ cross-section SEM images of the LLZTO-GC|Li during the first plating and stripping at 0.1 mA cm^{-2}. (b-g) In-situ cross-section SEM images of LLZTO-GC|Li during deposition and stripping at 0.4 mA cm^{-2} for 0.5 h, scale bar 30 μm.

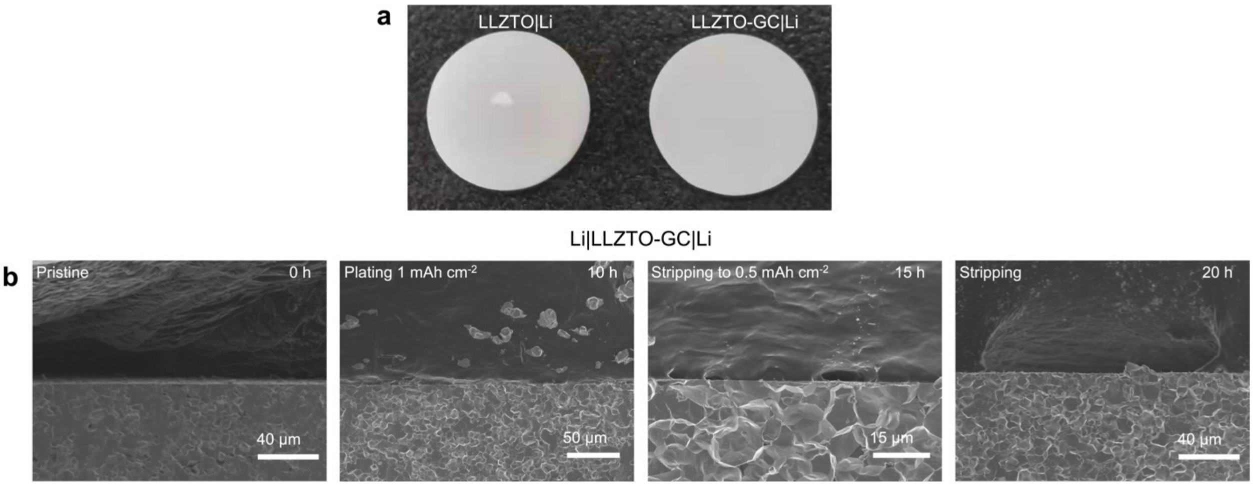

Figure S22. (a) Optical photograph of LLZTO after cycling in Li|LLZTO-GC|Li and Li|GC-LLZTO-GC|Li cells. (b) Ex-situ cross-section SEM images of the LLZTO|Li during the first plating and stripping at 0.1 mA cm^{-2}.

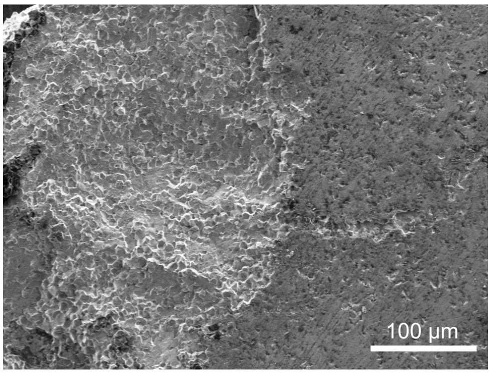

Figure S23. SEM image of LLZTO after the 1st cycle in a Li|LLZTO-GC|Li cell.

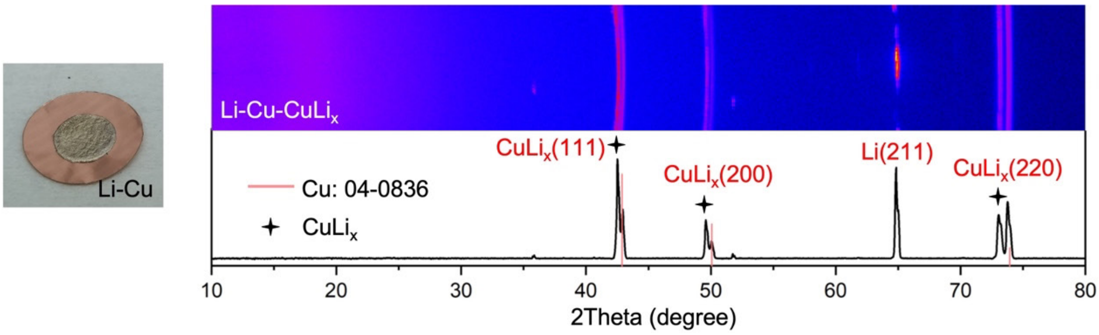

Figure S24. 2D-XRD and corresponding 1D-XRD for the texture of Li metal on Cu substrate.

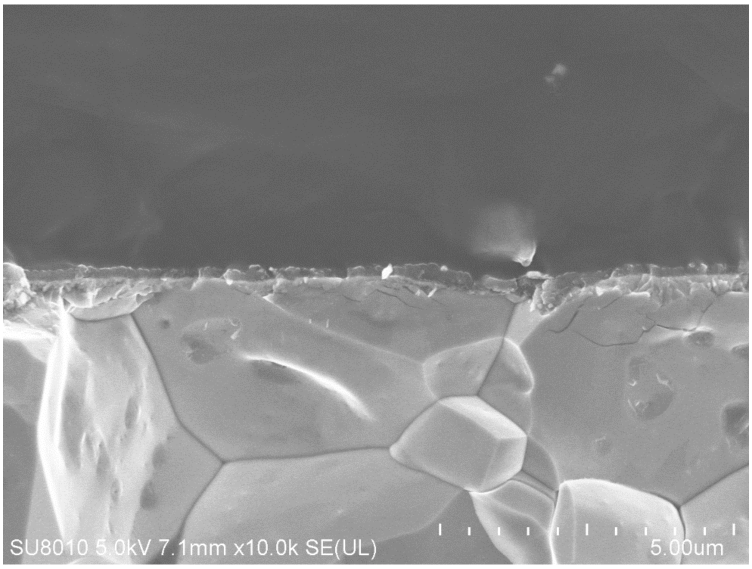

Figure S25. Cross-section SEM image of the as-prepared LLZTO-Cu|Li.

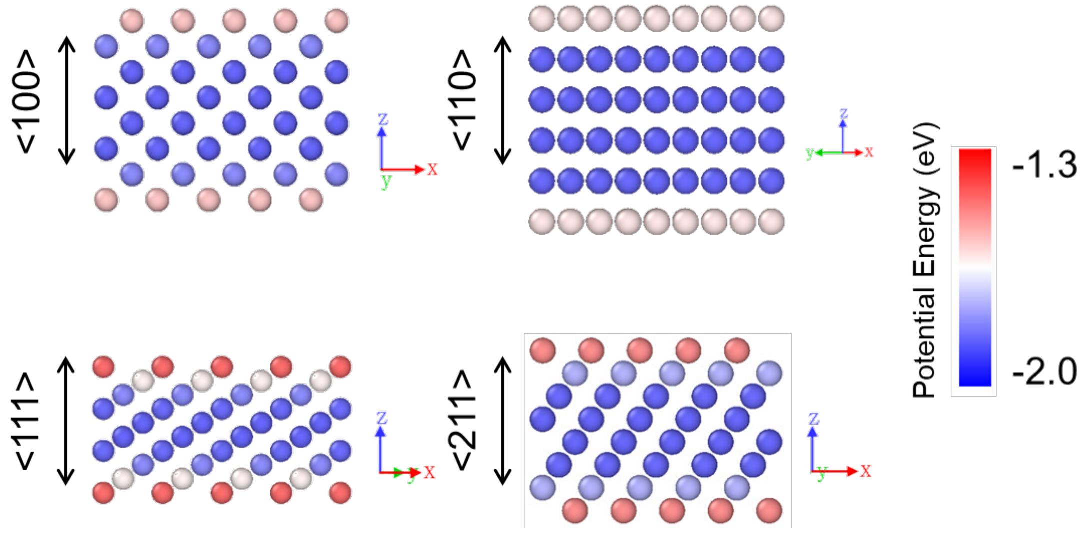

Figure S26. The arrangements of several common Li crystal planes.

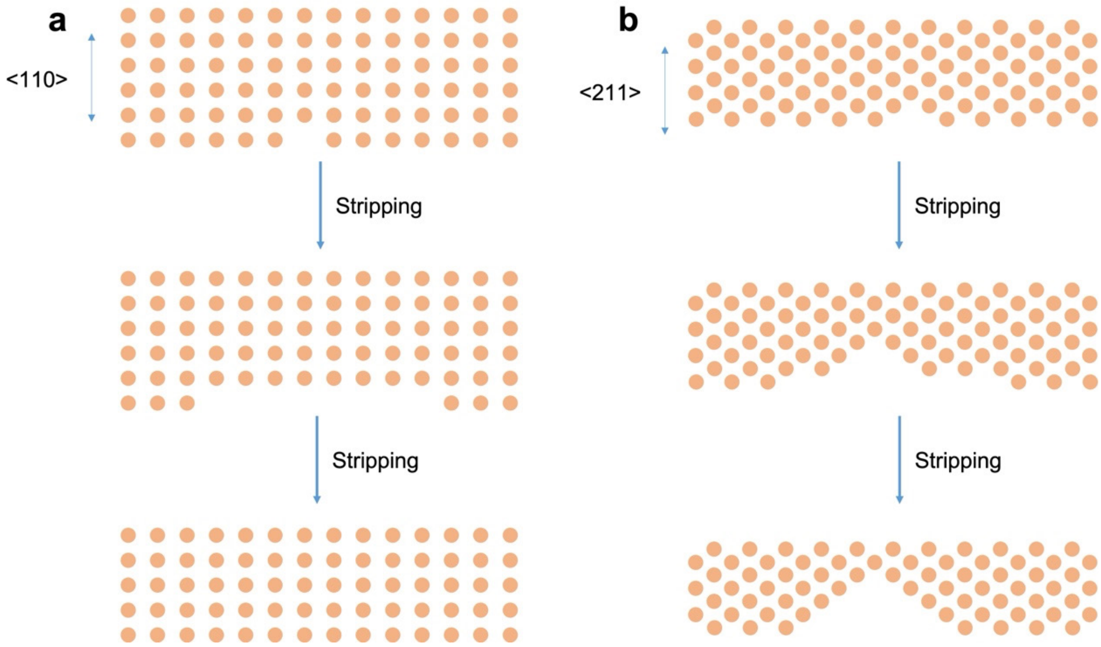

Figure S27. Schematic of the Li stripping behavior of (a) (110) and (b) (211) crystal planes.

.

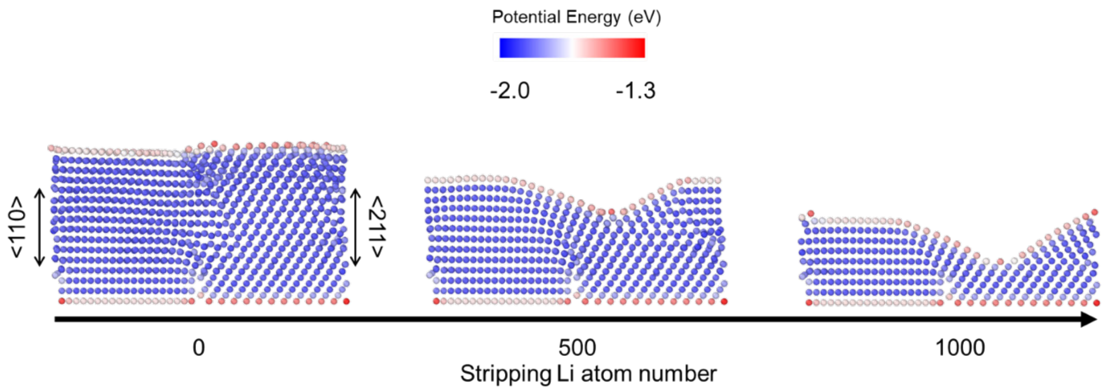

Figure S28. Dynamic Li stripping behavior of a polycrystalline Li interface composed of (110) and (211) crystal planes.

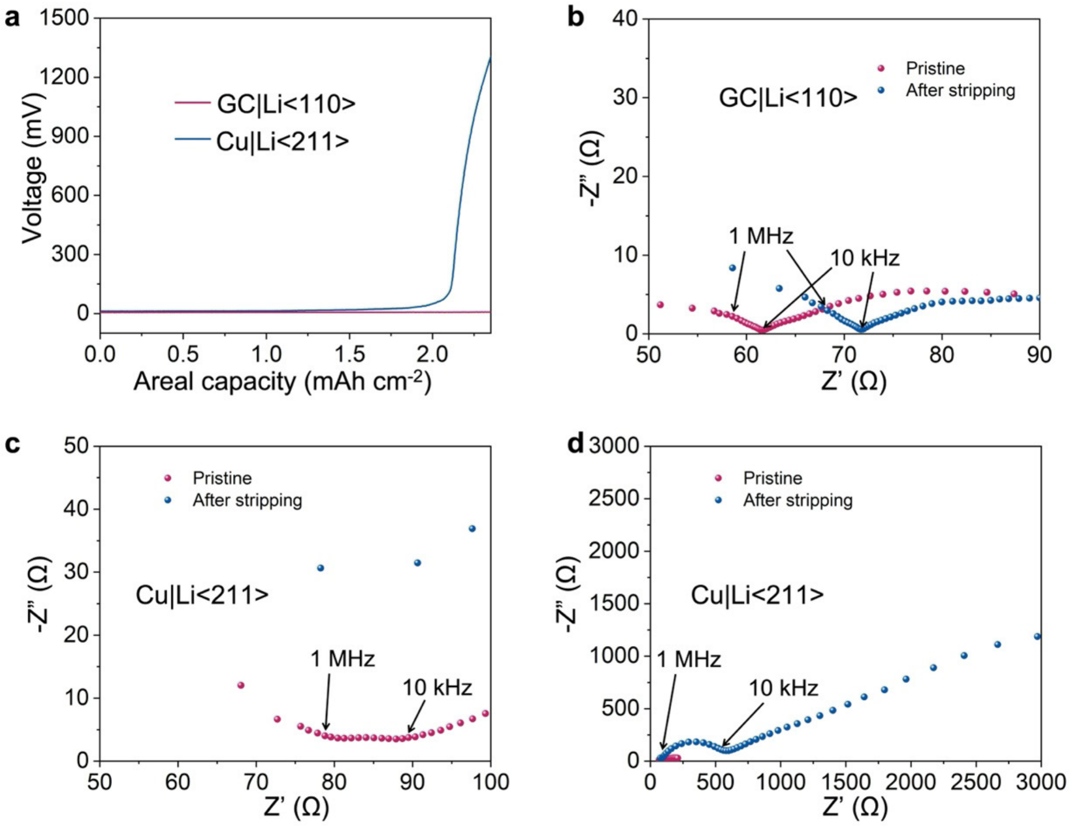

Figure S29. (a) Li stripping test of LLZTO-Cu|Li and LLZTO-GC|Li at 0.1 mA cm^{-2} with stripping capacity of 2.35 mAh cm^{-2} in Li|GC-LLZTO-Cu|Li and Li|GC-LLZTO-GC|Li cells. (b-d) EIS plot of Li|GC-LLZTO-Cu|Li and Li|GC-LLZTO-GC|Li cells before and after a Li stripping test.

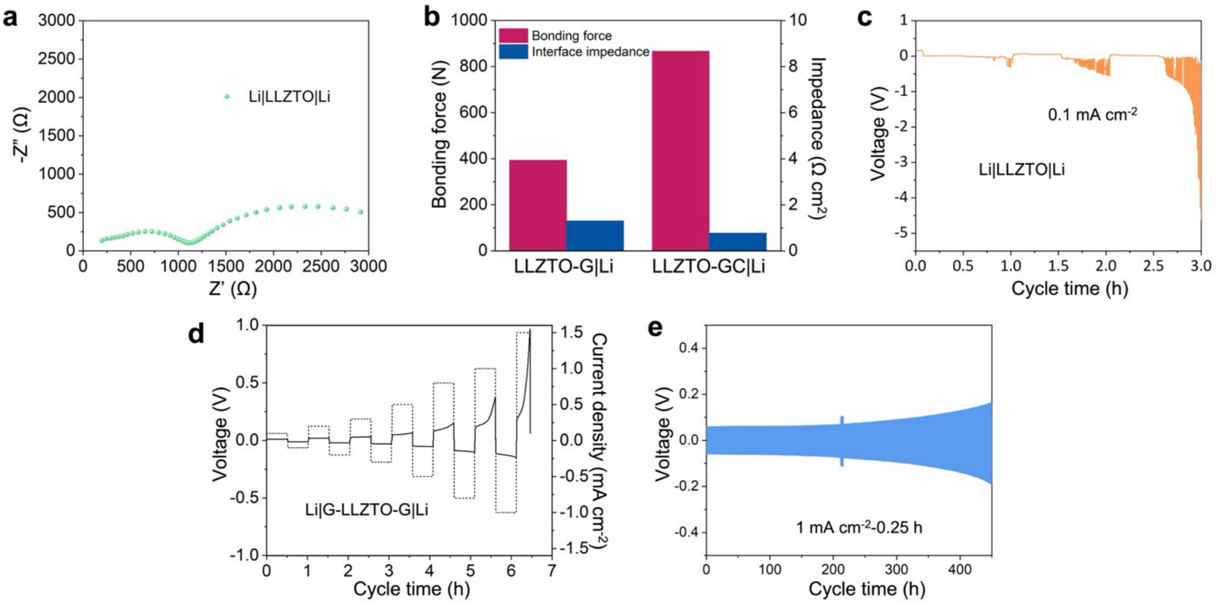

Figure S30. (a) EIS plot of a Li|LLZTO|Li symmetric battery. (b) Bonding force and interface impedance of LLZTO-GC|Li and LLZTO-G|Li. (c) Cycling performance of a Li|LLZTO|Li symmetric battery at 0.1 mA cm^{-2}. (d) Critical current density test with constant charge/discharge time (0.5 h) of a Li|G-LLZTO-G|Li cell. (e) Cycling performance of a Li|GC-LLZTO-GC|Li cell at 1 mA cm^{-2}-0.25 h.

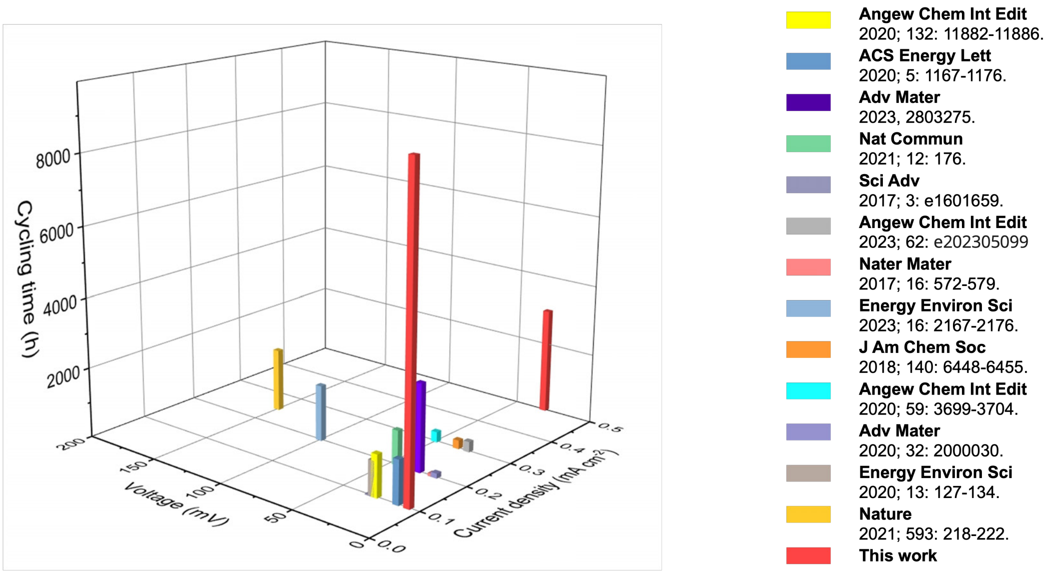

Figure S31. Electrochemical performance of Li|Li symmetric batteries with ISEs systems at room temperature reported in recent years.

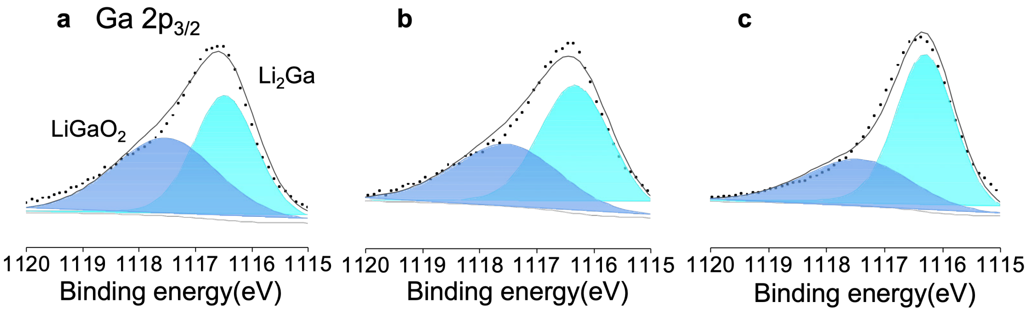

Figure S32. XPS analysis of the GC interface from LLZTO-GC|Li after reaction at (**a**) 200, (**b**) 250 and (**c**) 300°C.

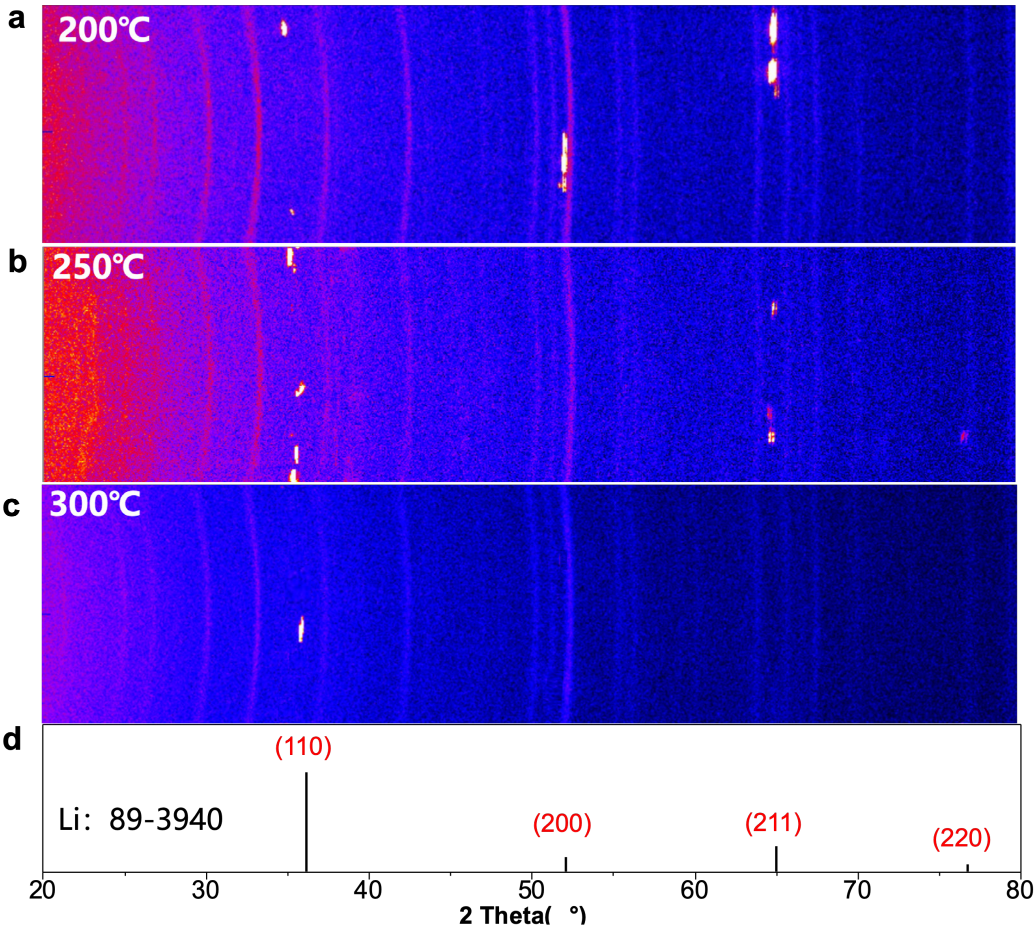

Figure S33. 2D-XRD patterns of LLZTO-GC|Li after reaction at (a) 200, (b) 250 and (c) 300℃. (d) Standard XRD pattern of Li metal.

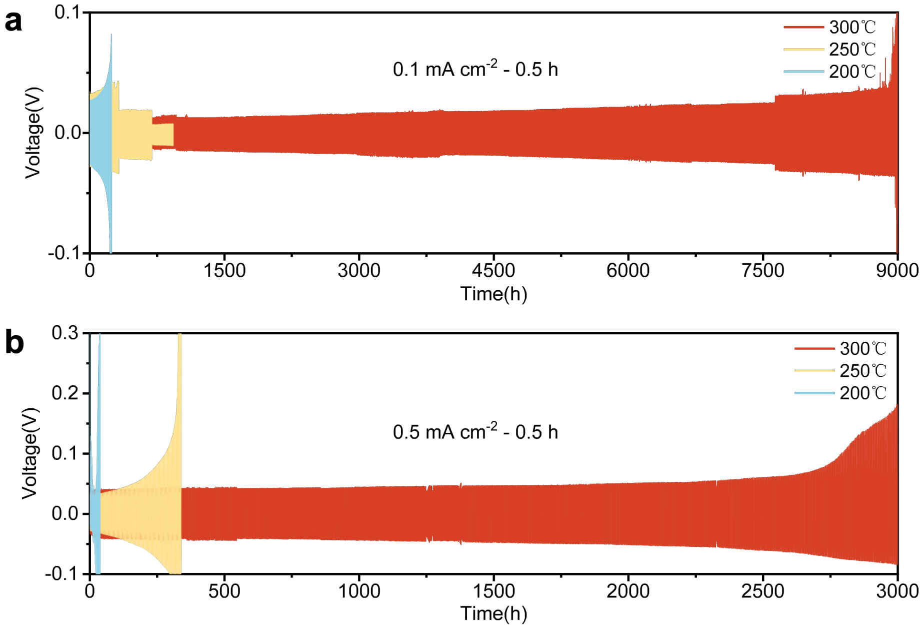

Figure S34. Cycling performance of Li|GC-LLZTO-GC|Li symmetric batteries under (a) 0.1 mA cm^{-2} and (b) 0.5 mA cm^{-2} and the LLZTO-GC|Li obtained after reaction at 200, 250 and 300°C.

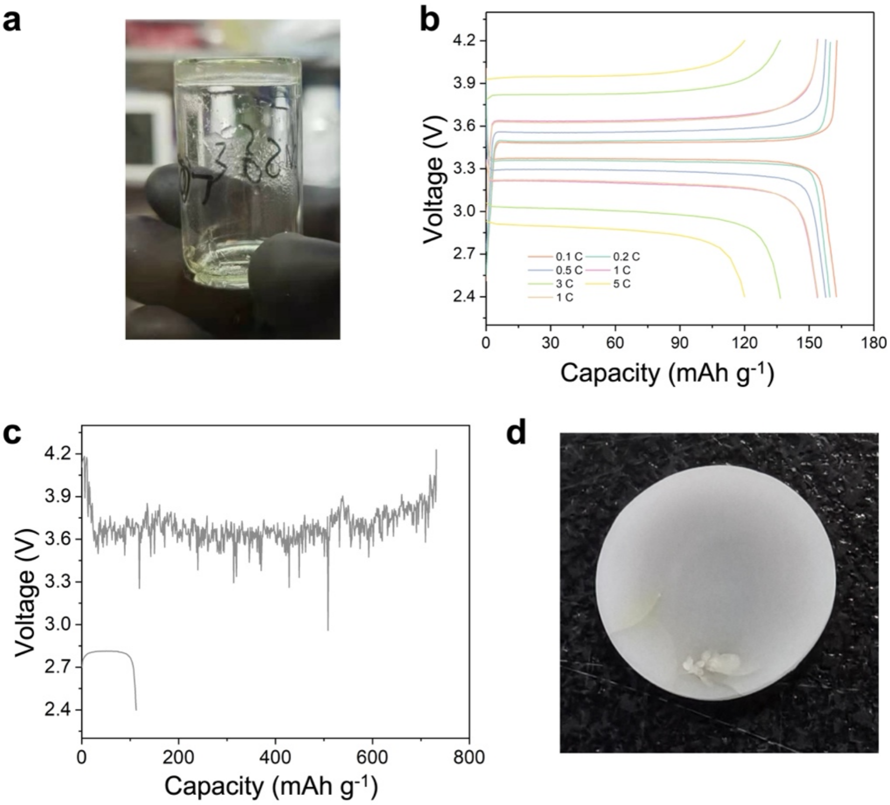

Figure S35. (a) Optical photograph of the succinonitrile-based electrolyte at room temperature. (b) Charge/discharge curves of LFP|LLZTO-GC|Li cells at different rates. (c) Charge/discharge curve of LFP|LLZTO-G|Li cells after 5 C shows the typical overcharge phenomenon. (d) Optical photograph of LLZTO in LFP|LLZTO-G|Li cells after rate test.

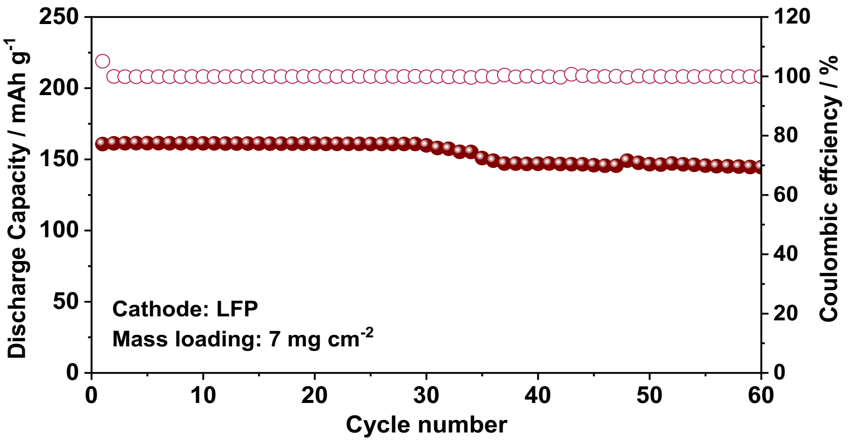

Figure S36. Cycling performance of all-solid-state LFP|LLZTO-GC|Li batteries at 0.1 C with a cathode mass loading of 7 mg cm^{-2} and an areal capacity of 1.22 mAh cm^{-2}. 1 C= 170 mA g^{-1}.

Supplementary Table

Table S1. The surface energy calculations of the main crystal planes of Li_2Ga.

Crystal plane	Surface energy (J m^{-2})
(001)	3.258994451
(021)	4.28013156387215
(100)	3.37289863889013
(110)	4.92307401432656
(111)	3.43886260341237
(131)	3.15793152697681

Table S2. Comparison of the electrochemical performance of LLZTO-based LFP||Li full cells

Interfacial layer	Rate (C)	Cycle lifespan	Capacity retention	Reference
an organic-inorganic gel interlayer	1	400	90.20%	*Mater. Today 2025, 87, 77*
HPIB	1	800	90.00%	*Adv. Mater. 2025, 37, 2415966*
N30	0.5	250	–	*Adv. Funct. Mater. 2025, e12943*
$Li_4(BH_4)_3I$/BN	0.1	90	78.91%	*Small 2025, e01766*
UW-LiMg	0.5	200	90.00%	*Adv. Sci. 2025, 12, 36, e04388*
polyoxanorbornene-based polymer interlayer	0.3	300	73.30%	*ACS Energy Lett. 2025, 10, 10, 4866–4871*
TfOH	2	500	81.00%	*Nat. Commun. 2024, 15, 9920*
Li_2O–LiOH–Li_3Sb	1	1000	80.00%	*Energy Environ. Sci., 2024, 17, 5819–5832*
Li-Sr-N	1	200	95.90%	*Adv. Funct. Mater. 2024, 34, 22*
ALD-Li_2O layer	1	600	84.00%	*Adv. Funct. Mater. 2024, 34, 4, 2306399*
Ag/LiF	2	3000	–	*Sci. Adv. 2022, 8, eabq0153*
PAA	1	500	92.30%	*Adv. Funct. Mater. 2022, 33, 10, 2213443*
**GC layer**	**3**	**6000**	**92%**	***This Work***
	**3**	**10000**	**62%**	***This Work***
	**1**	**3000**	**83%**	***This Work***

REFERENCES

1. Plimpton S. Fast parallel algorithms for short-range molecular dynamics. *J Comput Phys* 1995; **117**: 1-19.
2. Jiao J, Lai G, Zhao L*, et al.* Self-healing mechanism of lithium in lithium metal. *Adv Sci* 2022; **9**: 2105574.
3. Zhang L, Han J, Wang H*, et al.* Deep potential molecular dynamics: A scalable model with the accuracy of quantum mechanics. *Phys Rev Lett* 2018; **120**: 143001.
4. Stukowski A. Visualization and analysis of atomistic simulation data with OVITO–the Open Visualization Tool. *Model Simul Mater Sci Eng* 2010; **18**: 015012.
5. Momma K, Izumi F. *VESTA 3* for three-dimensional visualization of crystal, volumetric and morphology data. *J Appl Crystallogr* 2011; **44**: 1272-6.
6. Kresse G, Furthmüller J. Efficient iterative schemes for *ab initio* total-energy calculations using a plane-wave basis set. *Phys Rev B* 1996; **54**: 11169-86.
7. Kresse G, Furthmüller J. Efficiency of ab-initio total energy calculations for metals and semiconductors using a plane-

wave basis set. *Comput Mater Sci* 1996; **6**: 15-50.
8. Perdew JP, Burke K, Ernzerhof M. Generalized gradient approximation made simple. *Phys Rev Lett* 1996; **77**: 3865-8.
9. Blöchl PE. Projector augmented-wave method. *Phys Rev B* 1994; **50**: 17953-79.
10. Tan J, Ma L, Yi P*, et al.* Scalable customization of crystallographic plane controllable lithium metal anodes for ultralong-lasting lithium metal batteries. *Adv Mater* 2024; **36**: 2403570.
